# Intrinsic Viral Factors Are the Dominant Determinants of the Hepatitis C Virus Response to Interferon Alpha Treatment in Chimeric Mice

**DOI:** 10.1371/journal.pone.0147007

**Published:** 2016-01-14

**Authors:** Ran Chen, Michelle Kobewka, William Addison, Gerald Lachance, D. Lorne Tyrrell

**Affiliations:** 1 Department of Medical Microbiology and Immunology, Edmonton, Alberta, Canada; 2 Li Ka Shing Institute of Virology, Katz Centre for Health Research, Edmonton, Alberta, Canada; University of Montreal Hospital Research Center (CRCHUM), CANADA

## Abstract

**Background:**

Hepatitis C virus infection is a global health problem. New direct-acting antiviral agents have been recently approved. However, due to their high cost and some genotypes remaining difficult to treat, interferon-based therapy with pegylated interferon and ribavirin likely may remain a component of hepatitis C virus treatment for some patients. Unfortunately, pegylated interferon / ribavirin treatment achieved favorable outcomes in less than 50% of patients. Factors determining the outcome to pegylated interferon / ribavirin include both host and viral factors. It has been a major challenge to separate the host and viral factors in most *in vivo* systems.

**Aims & Methods:**

We used two hepatitis C virus strains from patients with different interferon-sensitivities and three hepatocyte donors, each with distinct interleukin 28B and interferon lambda 4 single nucleotide polymorphisms to investigate the contributions of viral and host factors to the response of hepatitis C virus to interferon treatment in chimeric mice.

**Results and Conclusions:**

We found that viral factors were the dominant factors in determining the interferon treatment outcomes in chimeric mice. Host factors, such as pre-treatment liver interferon-stimulated gene expression and single nucleotide polymorphisms near interleukin 28B and interferon lambda 4 coding regions, were less important determinants of the response to interferon in the chimeric mice than they were in patients. Our results also suggest that a complete immune system as seen in patients may be required for host factors such as single nucleotide polymorphisms near interleukin 28B / interferon lambda 4 and pre-treatment liver interferon-stimulated gene upregulation to have an effect on the interferon response.

## Introduction

Infection with hepatitis C virus (HCV) is a global health problem. Approximately 50% to 85% of HCV-infected people develop chronic hepatitis [1]. A combination of pegylated interferon alpha (pegIFNα) and ribavirin (RBV) has been used to treat patients with chronic HCV for more than a decade [2,3]. However, in about 50% of patients, the infection is not cleared with this therapy or the HCV relapses after treatment is completed [2,3]. The recent introduction of direct-acting antiviral agents (DAAs) has provided very important treatment options with significantly improved rates of viral clearance for individuals infected with HCV genotype 1, the most common genotype in North America and Europe [4–6]. However, the high cost of DAAs and some genotypes remaining difficult to treat, e.g. genotype 3, may present a challenge for the implementation of DAAs worldwide [6]. Therefore, pegIFNα and RBV therapy may remain a component of HCV treatment for some patients.

Both viral and host factors have been shown to be involved in determining interferon treatment outcomes in chronic hepatitis C (CHC) patients. In addition to non-genetic factors (e.g., age, alcohol, obesity, smoking), upregulation of pre-treatment intraheptic expression of certain interferon-stimulated genes (ISGs) has been shown to be a host factor associated with poor-response to IFN therapy [7,8]. In addition, the identification of polymorphisms in the human genome, especially the alleles of two single nucleotide polymorphisms (SNPs) near the IL28B coding region, *rs12979860* and *rs8099917* [9–11] as well as the most recently discovered IFN lambda 4 (*IFNL4)* SNP *rs368234815* [12,13], have improved our understanding of response and non-response and to predict the likelihood of a sustained virological response (SVR) to IFN therapy. Among viral factors, HCV genotypes are well accepted as the strongest predictor of IFN response. In addition, amino acid variations in the IFN-sensitivity-determining region (ISDR) within the NS5A protein [14,15], as well as at amino acid 70 or 91 in the HCV core protein [16,17] are predictors of SVR and non-virological response (NVR). Early evolution of HCV quasispecies, e.g., the degree of quasispecies’ complexity and diversity of hypervariable region 1 (HVR1), is another marker that is closely correlated with responsiveness to interferon therapy among CHC patients [18–21].

Assaying these host and viral factors in clinical situations is complex because each patient presents with a unique set of viral and host components that may interfere with his or her response to IFN. The first non-primate small animal model to support HCV infection, the severe combined immunodeficiency (SCID) / beige—Albumin (Alb) / urokinase-type plasminogen activator (uPA) mouse transplanted with human hepatocytes to create a chimeric human/mouse liver, was reported in 2001 [22]. One of the unique opportunities offered by this model is the ability to study both viral and host factors in the response of HCV to IFN therapy. Infections by HCV viruses of different genotypes can be studied in animals populated with human hepatocytes from a single donor. Conversely, a single strain of HCV can be used to infect chimeric mice transplanted with hepatocytes from different donors. The control of these variables allows us to determine the contributions of host and viral factors to the responsiveness of HCV infection to IFNα treatment.

In this study, our objective was to investigate the host versus viral factors that contribute to outcomes of IFN therapy in the chimeric mouse model. We studied the responses of two HCV strains with known IFN-response in patients to IFN treatment in mice transplanted with hepatocytes from three donors of different *IL28B/IFNL4* SNPs. We found that intrinsic virus factors, but not viral blockage of the host *JAK-STAT* pathway or known viral sequence variations, were key determinants of IFN therapy outcomes. Whereas host factors, such as polymorphism at the *IL28B/IFNL4* loci and upregulated pre-treatment levels of intrahepatic ISG expression, were not as important determinants of IFN therapy outcomes in chimeric mice, which lack an adaptive immune system, as they are in humans.

## Materials and Methods

### Generation of SCID/beige-Alb/uPA chimeric mice

All mice were housed and maintained under specific pathogen-free conditions according to Canadian Council on Animal Care guidelines. All animal experiments in this study were approved by the University of Alberta Animal Care and Use Committee: Health Sciences (Study ID: AUP00000348, Study title: Study of HAV, HBV, and HCV in the chimeric mouse model). All surgery was performed under isoflurane inhalation anesthesia, and all efforts were made to minimize suffering. We transplanted cryopreserved, commercially purchased human primary hepatocytes into SCID/beige-Alb/uPA mice as described previously [22]. In this study, 4 hepatocyte donors were used: donors Hu8063, Hu4109 and Hu8085 (purchased from CellDirect Inc, USA) and donor FLO (Celsis). Four and eight weeks after transplantation, we determined human hepatocyte repopulation levels by measuring human albumin in mouse serum using sandwich ELISA. Briefly, we performed the sandwich ELISA using goat anti-human albumin antibody (Cedarlane) as the capture antibody, goat anti-human albumin antibody conjugated with HRP (BETHYL) as the detection antibody and a TMB microwell peroxidase substrate system (KPL) for detection purposes.

### HCV infection and interferon treatment

We used chimeric mice with human albumin concentrations equal to or greater than 5,000μg/mL at 8 weeks post transplantation for the HCV infection studies. Chimeric mice received a single intravenous injection of human patient HCV-positive serum (approximately 10^5^ genome equivalence). The collection and use of human sera in this study were approved by the Health Research Ethics Board, University of Alberta (Study ID: Pro00001040, Study Title: Blood Samples as a source of Hepatitis B or Hepatitis C viruses for studies in an animal model of HBV or HCV). Each patient has given written informed consent to donate blood to be used in this study.

We administered exogenous human IFNα subcutaneously (s.c.) for 14 days at 1,350 IU/g body weight/day using human IFNα-2b (INTRON® A, MERCK). Six hours after the last IFNα injection, we euthanized the mice and collected samples/tissues. We gave the same volume of saline to chimeric mice in the control group. For groups treated with pegylated human IFNα-2b (Pegasys, Roche), we administered 875ng once a week by s.c. injection. After 4 weeks, we discontinued treatment and monitored the animals for relapse for 2 weeks before termination.

### Tissue dissection

We terminated the mice by cervical dislocation and excised the livers, which we cut into small pieces and snap froze in liquid nitrogen or fixed in 10% formalin for further histological and molecular analyses. We isolated total RNA and genomic DNA with TRIZOL (Invitrogen) according to the manufacturer’s recommended protocol. We isolated both total RNA and genomic DNA from the same liver section sample.

### Human chimerism measurement

We determined the average human cell content of the chimeric liver using the TaqMan Copy Number Reference Assay (Applied Biosystems 4401613), which targets the single copy human RNase P gene. We took chimeric mouse liver that had been frozen in liquid nitrogen and powdered it using a mortar and pestle in liquid nitrogen. We isolated highly purified genomic DNA from aliquots of the powder by Proteinase K digestion, phenol and chloroform extraction, ethanol precipitation and spermine precipitation [23]. We constructed a standard curve for the TaqMan assay using known mixtures of human and mouse DNA (Promega G3041 and G3091).

### HCV titer measurement

We extracted HCV RNA from 30μL mouse serum samples using a High Pure Viral Nucleic Acid kit (Roche). We dried the extracted RNA samples in by vacuum centrifugation for 1.5 hours, and performed reverse transcription using HCV specific reverse primer (S1 Table) and ThermoScript™ reverse transcriptase (Invitrogen). We performed real-time quantification PCR on an ABI 7900 Real Time PCR machine with TaqMan chemistry using HCV specific primers and probe (S1 Table). We amplified known amounts of cloned HCV genomic cDNA in parallel to establish a standard curve for quantification and determined PCR efficiency using the slope of the standard curve. We determined viral load using Applied Biosystems SDS Software 2.3 (Applied Biosystems). We quantified intrahepatic HCV RNA levels in each chimeric mouse liver by loading equal mass amounts of total RNA.

### Analysis of human gene expression by RT-real time PCR

To determine expression levels of genes related to IFN signaling in human hepatocytes, we designed specific oligos to recognize human transcripts and not cross-react with murine genes using Primer Express 3.0 (Applied Biosystems) and validated them by RT-real-time PCR using intrahepatic total RNA isolated from Balb/c mice treated with poly I:C (Sigma). We performed reverse transcription using 2μg total RNA with random hexamer primers (Invitrogen) and M-MLV (Invitrogen). We performed gene expression realtime PCR was using an ABI 7900 Real Time PCR system and ran each target in duplicate with Taqman 2X PCR Universal Master Mix in a 10μL reaction. We normalized transcript levels of each gene relative to the human HPRT1. The sequences of human specific primers and probes are listed in S1 Table.

### SNP Genotyping

Genotying was performed using custom-designed TaqMan assays [12] (S1 Table) with Genotyping Master Mix (ABI) on the ABI 7900H using recommended standard conditions.

### Next-generation sequencing

Total RNA was extracted from mouse serum (pooled with 3–5 mouse samples in each group with the same treatment) and human patient serum (used as inocula for the mice) samples (S2 Table) using QIAamp viral RNA Mini kit (Qiagen), and cleaned up using RNeasy Mini kit with RNase-free DNase set (Qiagen). First strand cDNA was generated with Superscript II Reverse Transcriptase (Invitrogen) using random primers. Second strand cDNA was made using DNA Pol I (Invitrogen). Double stranded (ds) cDNA fragments were cleaned up using the Qiaquick PCR Purification kit (Qiagen) and eluted in 40μL ultrapure water. cDNA fragments were sheared with Covaris-S2 to average size of 400bp and Illumina library was constructed using Tru-seq protocol (NEBNext kit). Libraries with equal amount (samples 1, 2, 3 in S2 Table were pooled together and samples 4, 5, 6 in S2 Table were pooled together for another capture run) were pooled at total amount of 1.2 μg for each capture reaction using a probe-capture based protocol [24], and optimized for enriching low abundant HCV sequences with a probe set designed according to HCV reference genomic sequences (GI numbers: 157781216, 157781214, 157781212, 157781210, 157781208, and 22129792). HCV 3' UTR region was excluded due to high density of repeats. Libraries after capture were pre-amplified and proceeded to Illumina MiSeq sequencing using a 150 cycles' kit (paired end reads of 2X75bp). The paired-end reads were aligned against HCV reference genomic sequences using bowtie 2 [24], and mapping ratio, average depth, and coverage were obtained based on the alignment results to the best hits. Variation calling was further performed with samtools package and python scripts using stringent quality filter of Q30 to generate summary tables of nucleotide changes along viral genome, as well as amino acid changes in all protein coding genes. Specific regions of HCV core, NS5A and E2 genes were extracted out for further analysis.

### Statistics

We performed statistical analyses using Prism software for Mac OS X version 5.0. Data are expressed as means ± standard deviation (SD) or medians with ranges where appropriate. We used one-way ANOVA tests or t-tests for nonparametric pairwise comparisons. *P*-values < .05 are considered significant.

## Results

### Role of viral factors in determining exogenous human IFNα treatment outcomes in HCV-infected chimeric mice

We selected two HCV strains of different genotypes (Table 1), genotype (gt) 1a and gt2b, from two CHC patients prior to their treatment with pegIFNα and ribavirin. One HCV strain, HCVgt1a, was from a known null-responder, and a second HCV strain, HCVgt2b, was from a patient who had responded to pegIFNα/RBV therapy. The HCVgt2b strain was naïve for IFN treatment, i.e. the virus was isolated before the patient received IFN treatment.

**Table 1:** Two HCV clinical isolates with distinct sensitivities to IFN therapy.

HCV strain	Genotype	Patient response to IFN therapy
HCVgt1a	1a	Null responder
HCVgt2b	2b	SVR

In order to exclude the host variable, we produced all chimeric mice in this part of the study with human hepatocytes derived from a single donor, Hu8063. We first evaluated the ability of the two HCV strains to infect the chimeric mice to rule out the possibility that different response to IFN could be attributed to differences in their ability to sustain a chronic infection. We infected chimeric mice with each HCV strain for 7 weeks and monitored serum viremia. For both strains, HCV serum titers reached comparable levels 2 weeks post infection (p.i.) and remained at relatively constant levels for an additional 5 weeks (S1 Fig).

**Fig 1:**
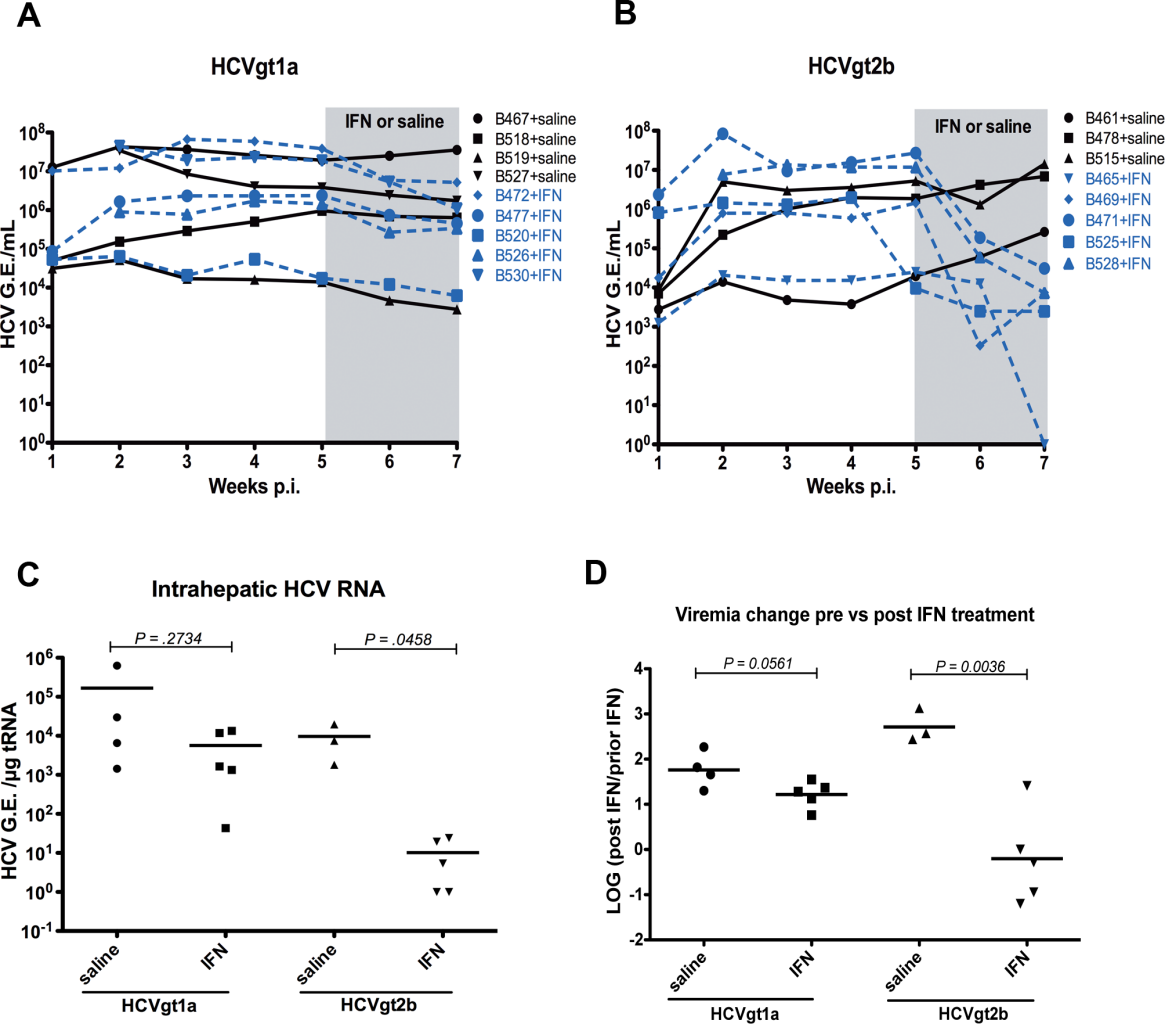
Responses of the two HCV strains to IFNα in mice populated with a single donor. We infected chimeric mice produced with a single hepatocyte donor, Hu8063, with 2 HCV strains for 5 weeks. We then subcutanously injected exogenous human IFNα-2b daily at 1,350 IU/gram for 14 days. We treated control chimeric mice with saline. We took sera from the mice weekly in order to measure viremia. Six hours after the last IFNα injection, we terminated the mice and measured intrahepatic HCV load. Data for mice treated with saline are represented by black solid lines and data for mice treated with IFNα are represented by blue dashed lines in **A** (HCVgt1a viremia titer) and **B** (HCVgt2b viremia titer). Each line in **A** and **B** represents a single mouse. The period of human IFNα/saline treatment is shaded in gray. A comparison of intrahepatic HCV RNA levels for different treatment groups at week 7 p.i. is shown in **C**. *P*-values in **C** were calculated using unpaired t-tests.

We next examined the responses of chimeric mice infected with each of the HCV strains (Table 1) to exogenous human IFNα treatment. Two groups of chimeric mice were infected, one group (9 mice) with HCVgt1a strain and another group (8 mice) with HCVgt2b strain, for 5 weeks to reach stable levels of viremia. We divided each infected group in two subgroups: one subgroup (5 mice) received exogenous human IFNα treatment and a control subgroup (3 to 4 mice) received saline. We measured viremia levels during the course of IFNα/saline treatment. Data in Fig 1 show that the two HCV strains in hepatocyte donor-matched chimeric mice responded to human IFNα treatment differently. Strain HCVgt1a exhibited minimal response (< 1 log10 reduction) after 2 weeks of IFNα treatment (Fig 1A). However, strain HCVgt2b showed more than 3 log10 decline in serum HCV RNA levels after 2 weeks of IFNα treatment (Fig 1B). The viremia of one mouse infected with HCVgt2b strain became undetectable after this short course of therapy (Fig 1B). To rule out the possibility that the change in virus levels was due to a decrease in the number of human hepatocytes in the chimeric liver, we measured the level of human albumin, a marker of human chimerism, in the serum of each chimeric mouse by enzyme-linked immunosorbent assay (ELISA). During the course of infection and human IFNα treatment, we observed no significant decrease in the human hepatocyte content of the chimeric livers based on serum human albumin levels (S2 Fig). In addition, at the end of the experiment, we measured the number of HCV RNA copies in the livers of infected chimeric mice. Chimeric mice infected with the HCVgt2b strain showed a significant decrease in intrahepatic HCV levels after human IFNα treatment, whereas chimeric mice infected with HCVgt1a did not (Fig 1C), which mirrored the effect of human IFN on serum levels in these mice (Fig 1D).

To further confirm the significant effect of viral factors on HCV responses to IFN therapy, we infected chimeric mice with additional HCV isolates taken from patients with various response outcomes to pegIFN and RBV treatment. In order to obtain a better comparison between chimeric mice and patients, we used human pegIFNα rather than IFNα in this experiment. We did not use RBV because of its toxic effect in chimeric mice. Chimeric mice stably infected with HCV isolates were treated with a weekly subcutaneous (s.c.) injection of human pegIFNα for 4 weeks. Followed by a 2-week treatment-free period, the mice were euthanized. We monitored the change of viremia levels throughout the experimental period. Our results in Table 2 show a good correlation between the HCV responses to human pegIFNα in chimeric mice and the responses observed in patients. For example, there are 7 mice that were produced with the same human hepatocyte donor (Hu8085) shown in Table 2. Four of them received 3 different isolates of HCV gt1, while the other 3 received 2 different isolates of HCV gt2, yet the response to pegIFNα treatment always correlated with the response in the corresponding patient. These results support the critical role of viral factors in predicting responses to IFN therapy.

**Table 2:** Correlation of HCV response to pegIFN treatment in chimeric mice with the response of patients to the pegIFN and RBV therapy*.

Mouse ID	Hepatocyte donor	HCV isolate	HCV genotype	Response in mouse	Response in patient
B1369	Hu8063	UA110	1a	Nonresponder	No
B1379	Hu8063	UA110	1a	Nonresponder	No
B1242	Hu8085	UA164	1	Nonresponder	No
B1270	Hu8085	UA164	1	Nonresponder	No
B1305	Hu8085	UA174	1a	Nonresponder	No
B1310	Hu8085	UA176	1	Partial responder	No
B1268	Hu8085	UA131	2	Responder	Yes
B1488	Hu8085	UA166	2	Responder	Yes
B1490	Hu8085	UA166	2	Responder	Yes
B1478	FLO	UA141	1a	Responder	Yes
B1486	FLO	UA141	1a	Responder	Yes
B1485	FLO	UA143	1a	Responder	Yes
B1483	FLO	UA159	5a	Responder	Yes
B1482	FLO	UA177	3a	Responder	Yes
B1462	FLO	UA178	1a	Responder	Yes
B1471	FLO	UA178	1a	Responder	Yes

* Individual chimeric mouse (column “**Mouse ID**”) produced with human hepatocytes (column “**Hepatocyte donor**”**)** were infected by HCV isolates (column “**HCV isolate**”) of various genotypes (column “**HCV genotype**”). HCV response to pegIFN treatment in each chimeric mouse (shown in column “**Response in mouse**”) was compared with the response of patients to the pegIFN and RBV therapy (shown in column “**Response in patient**”).

Among viral factors, amino acid variations at amino acid 70 or 91 in the HCV core region [16,17] as well as in the ISDR within the NS5A region [14,15] are predictors of treatment outcomes. In addition, diversity of HVR1 at E2 region is another marker that is closely correlated with responsiveness to interferon therapy among CHC patients [18–21]. We examined the two original HCV strains used in this study (Table 1) as well as the serum samples from mice infected by the two HCV strains with saline or IFN treatment using next-generation sequencing technology. Mouse samples of each group given the same inoculum and the same treatment (S2 Table) were pooled for pre-capture cDNA library construction. cDNA fragments in the library were further pooled (S2 Table) and captured with a HCV-specific probe set to enrich low abundant HCV sequences.

Based on the reads distribution plots of the HCVgt1a genome (S3 Fig), we have over 99% genome coverage from all three samples (sample #1–3 in S2 Table) with more than 200X average read depth. Relatively low genome coverage and read depth was observed against HCVgt2b sequences (S4 Fig) in all three samples (samples #4–6 in S2 Table). This could be due to relatively low similarity between probe sequences and the HCV target sequences or limited HCV RNA in serum after treatment. A nearly full genome of HCV genotype 2-like sequence (>90% of whole genome) (S3 Table) was assembled using SOAPdenovo from pooled reads of test 2 (S2 Table). This novel HCV genomic sequence has about 75% similarity to the reference genome (GI: 157781212) used for probe design.

According to the variation calling results (S4 Table), amino acid substitutions at aa 70 or 91 in the HCV core region and in the ISDR (aa 2209–2248) within the NS5A region, as well as amino acid (aa384-411) and nucleotide (nt1150-1233) diversity of HVR1 at E2 region were analyzed in all three HCVgt1a samples (S2 Table). As shown in Table 3, we did not observe specific amino acid substitutions at aa 70 or 91 in the HCV core region, e.g. aa R70Q, aa R70P or aa L91M, which were associated with non-responders in genotype 1b HCV infection as previously reported [16,17]. Although decreased occurrence of mutations at HCV NS5A ISDR was noticed in mouse samples infected with HCVgt1a (sample #2 in S2 Table) in comparison to the original human isolate (sample #1 in S2 Table), the number of mutations in mouse samples treated with saline (sample #2 in S2 Table) versus treated with IFN (sample #3 in S2 Table) remained unchanged (4 significant mutations in each sample, Table 3), whereas, published studies using large number of human samples showed that at least 4 amino acid changes within ISDR of NS5A in HCV genotype 1 were more sensitive to IFN [14,15]. Since individual mouse samples in each group of treated mice were pooled to improve cDNA library construction efficiency, the nucleotide / amino acid diversity at HVR1 region of E2 gene was analyzed based on the number of mutations of individual location. Decreased diversity at HVR1 region was observed at nucleotide level, which is consistent with previous studies [18–20], indicating a potential selection pressure on the HCV HVR1 region by IFN treatment.

**Table 3:** Amino acid or nucleotide variations in HCVgt1a and derived mouse samples treated with saline or IFN ^a^.

HCV gene	Location	Amino acid or nucleotide variations		
		HCVgt1a (human)	HCVgt1a (mouse, saline)	HCVgt1a (mouse, IFN)
**Core**	aa 70	8 (5.16%)^b^	3 (9.3%)^b^	2 (4.76%)^b^
	aa R70Q or R70P?	2 (0.11%)^b^	0	0
	aa 91	1 (0.67%)^b^	2 (8.40%)^b^	1 (3.23%)^b^
	aa L91M?	0	0	0
**NS5A (ISDR)**	aa 2209–2248	40 (8)^c^	40 (6)^c^	40 (4)^c^
**E2 (HVR1)**	nt 1150–1233	38^d^	12^d^	7^d^
	aa 384–411	17 (12)^c^	6 (4)^c^	4 (4)^c^

*a*. The results presented in this table are calculated based on the data in S4 Table.

*b*. Total number of substitutions (based on the reference sequence) at aa 70 and 91 of HCV core region was counted and shown in the table. The percentage of substitutions at given location, which is calculated as total number of substitutions / total number of reads of the location, was indicated in brackets.

*c*. Total number of substitutions (based on the reference sequence) in the ISDR or HVR1 region was shown. The number of significant substitutions was shown in brackets. The significant substitution is defined as percentage of substitutions greater than 5%.

*d*. Results were shown as the total number of changed nucleotides within HVR1 region in comparison to the reference sequence.

### Role of viral blockage of host IFN signaling in determining the sensitivity of HCV to exogenous human IFNα treatment in chimeric mice

The two HCV strains (Table 1) used in this study exhibited different responses to exogenous human IFNα treatment in chimeric mice produced with identical donor cells. It is known that HCV infection can block the IFN innate response in HCV-infected cells [25]. We thus hypothesized that the different sensitivities of the two HCV strains to human IFNα was possibly caused by differences in each strain’s ability to interfere with IFNα signaling, e.g., via the *JAK-STAT* pathway. We terminated chimeric mice infected with each of the two HCV strains (Table 1) 6 hours after we administered the last human IFNα injection, and collected the chimeric mouse livers. We assayed activation of the *JAK-STAT* pathway by measuring the changes in expression levels of a number of human-specific intrahepatic ISGs using RT-real time PCR. We compared the changes in IFN-induced ISGs to those in age-matched, hepatocyte donor-matched, uninfected chimeric mice that had been treated with saline. We observed significant upregulation of most of the human ISGs tested in uninfected mice treated with exogenous IFNα (S5 Fig), indicating that the chimeric mouse model used in this study can respond to human IFNα treatment. We also observed upregulation of ISGs in both sets of chimeric mice infected with the two HCV strains in response to human IFNα treatment (S5 Fig), however, the upregulated ISGs induced a significant decline in HCV viremia only in chimeric mice infected with the HCVgt2b strain (Fig 1).

**Fig 2:**
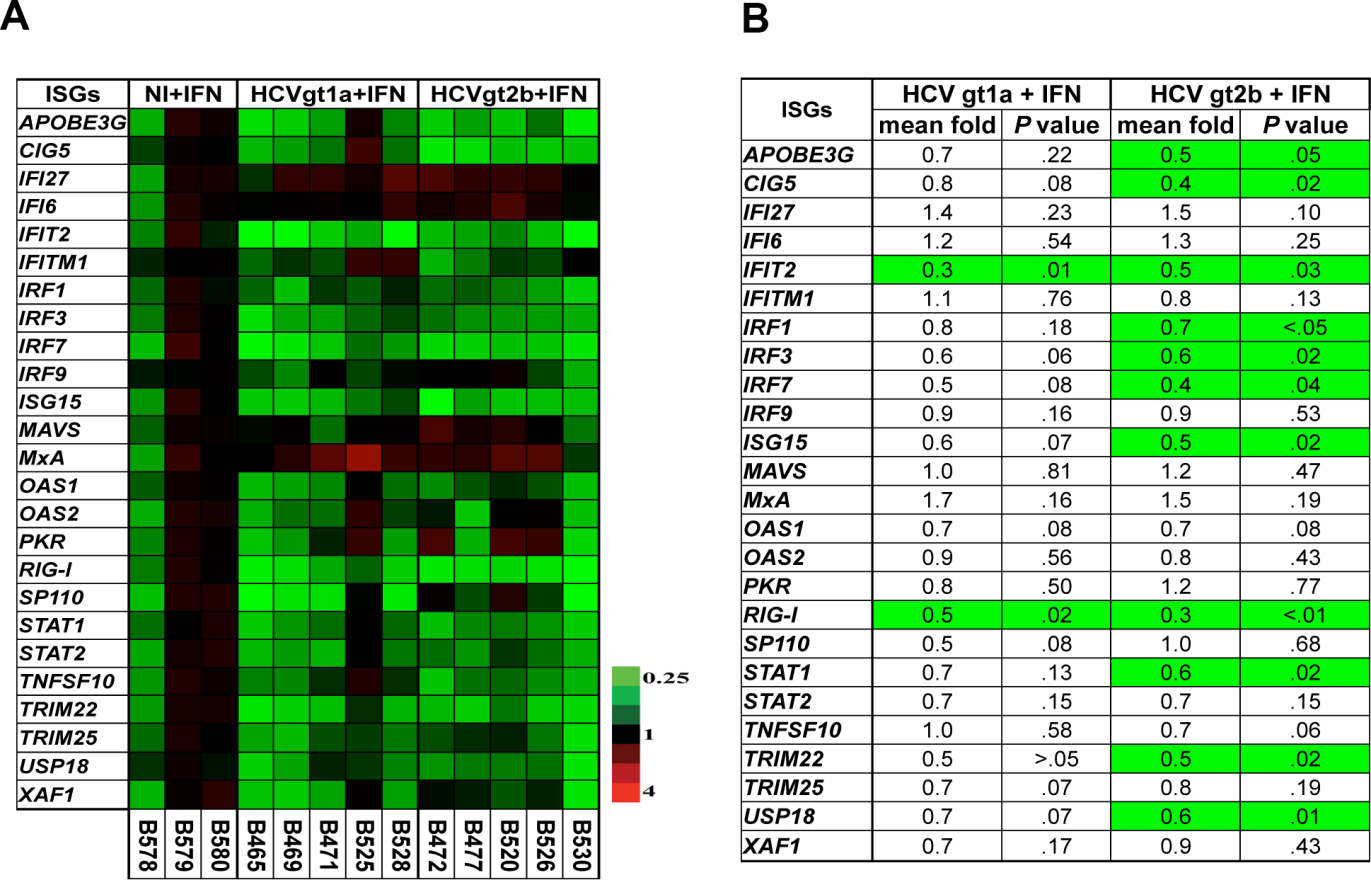
HCV interference with ISG expression upon IFNα treatment in chimeric mice. We analyzed the differences in expression levels of intrahepatic human ISGs among uninfected (NI), IFNα-treated chimeric mice and IFN-treated chimeric mice infected with either HCVgt1a or HCVgt2b. The uninfected IFNα-treated animals were set as the baseline for comparison. Each column in the heat map (**A**) represents the results in a mouse in each group. *P*-values in **B** were calculated using unpaired t-tests. In **B**, significantly (*P <* .05) upregulated and downregulated expression of ISGs compared to the uninfected IFNα-treated animal controls is indicated, respectively, by red and green highlighting in the tables. No highlighting indicates no significant difference in comparison to the uninfected IFNα-treated control group.

In order to compare the suppressive effect of the two HCV strains on the *JAK-STAT* pathway, we measured human ISG expression levels in chimeric mice infected with the two HCV strains after human IFNα treatment and compared these levels to the levels of ISG expression in uninfected control chimeric mice treated with human IFNα (Fig 2). We hypothesized that differences in sensitivity to IFNα treatment were determined by differences in the ability of HCV strains to interfere with the *JAK-STAT* pathway. If this was correct, we would expect to see more inhibition of human ISG expression in mice infected with the IFN-nonresponsive HCVgt1a strain than in mice infected with the IFN-responsive HCVgt2b strain. Surprisingly, results in Fig 2B show that the ISG expression in response to exogenous IFNα treatment in chimeric mice infected with the HCVgt2b strain was not significantly different than the ISG expression in mice infected with the HCVgt1a strain. In fact, there were more ISGs with significantly lower levels of mRNA present in chimeric mice infected with HCVgt2b than in mice infected with HCVgt1a (Fig 2B). Nevertheless, this finding supports the observation that intrinsic viral factors are crucial determinants of responses to IFN treatment for HCV infection, as opposed to the ability of the virus to block host IFN signaling.

### Effect of host pre-treatment ISG expression level on HCV responses to human IFNα treatment in chimeric mice

In CHC patients, high ISG expression before therapy is initiated is associated with poor response to pegIFNα/RBV treatment [7,8]. Conversely, ISG expression in liver biopsies of SVR patients is usually comparable to that of healthy adults prior to treatment [7,8]. In light of these findings, we studied pre-treatment ISG expression in both uninfected chimeric mice and chimeric mice infected with IFN nonresponsive and responsive HCV strains.

**Fig 3:**
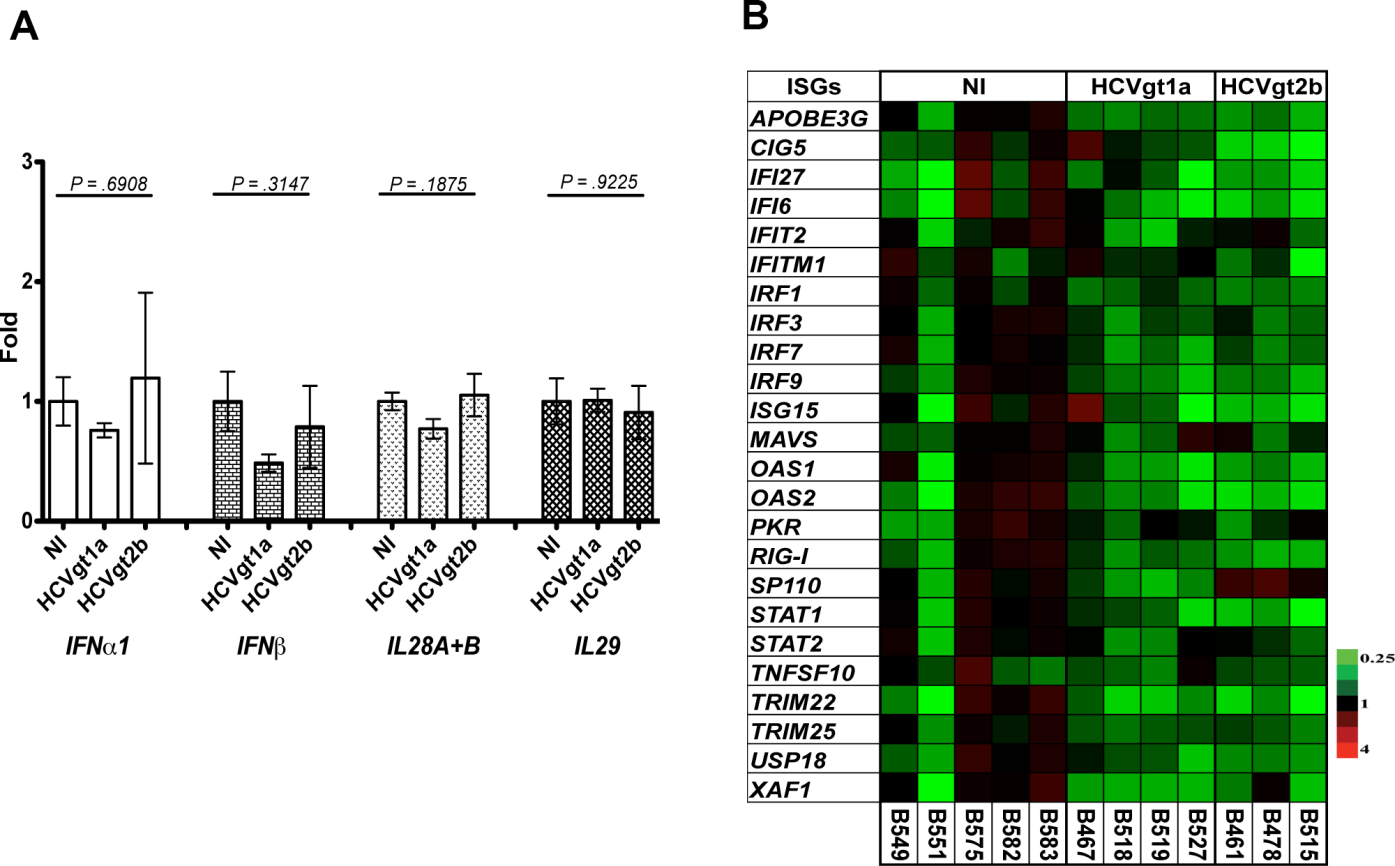
Intrahepatic IFN or ISG expression in mice infected with the two HCV strains. We infected chimeric mice produced with hepatocytes from donor Hu8063 with either HCVgt1a or HCVgt2b. Seven weeks p.i., we euthanized the mice and determined the expression levels of human-specific endogenous IFN genes (**A**) and ISGs (**B**) relative to a house-keeping gene HPRT-1 using the 2^-ΔΔCt^ method. In uninfected donor-matched chimeric mice, the mean value of each gene mRNA level was normalized to 1. Data in **A** are mean ± the standard error of the mean (SEM); n = 3 to 5. We determined *P*-values using a one-way ANOVA calculation. In **B**, each column represents an experimental mouse. Increased and decreased expression of specific genes compared to the uninfected control group is shown in red (Fold >1 to ≥4) and green (Fold <1 to ≤0.25), respectively, whereas black indicates no change (Fold = 1).

We terminated the uninfected animals and the chimeric mice infected with the two HCV strains (Table 1) and collected chimeric liver tissues. We measured mRNA levels of human type I IFNs (*IFNα1 and IFNβ*), type III IFNs (*IFNλ1–3*) and human-specific ISGs in chimeric mice infected with the two HCV strains using RT- real time PCR and compared them to mRNA levels of the same genes in the uninfected chimeric mice. We found that long-term infection of the two HCV strains in hepatocyte donor-matched chimeric mice did not induce any significant upregulation of endogenous human IFN (Fig 3A) or ISG (Fig 3B) expression in comparison to the uninfected controls. This result indicates that no upregulated IFN response was present during the chronic phase of HCV infection in the chimeric mouse model, a result consistent with the findings in tissue culture systems infected with a HCV virus, JFH-1 [26,27].

### The role of host IL28B polymorphisms in determining human IFNα treatment outcome in HCV-infected chimeric mice

Polymorphisms of *rs12979860*, *rs8099917 and rs368234815*, two SNPs upstream of the IL28B coding region and one upstream of *IFNL4* coding region, are characteristic host factors that predict responses of CHC patients to IFN treatment [9–13,28,29]. At SNP *rs12979860*, *CC* is considered the responder allele, whereas *CT* and *TT* are poor responder alleles [10,11]. At SNP *rs8099917*, *TT* is the responder allele and *GG* and *GT* are the poor responder alleles [9,10]. At SNP *rs368234815*, *TT/TT is associated with SVR and ΔG/ΔG and ΔG/TT are correlated with response failure* [12].

**Fig 4:**
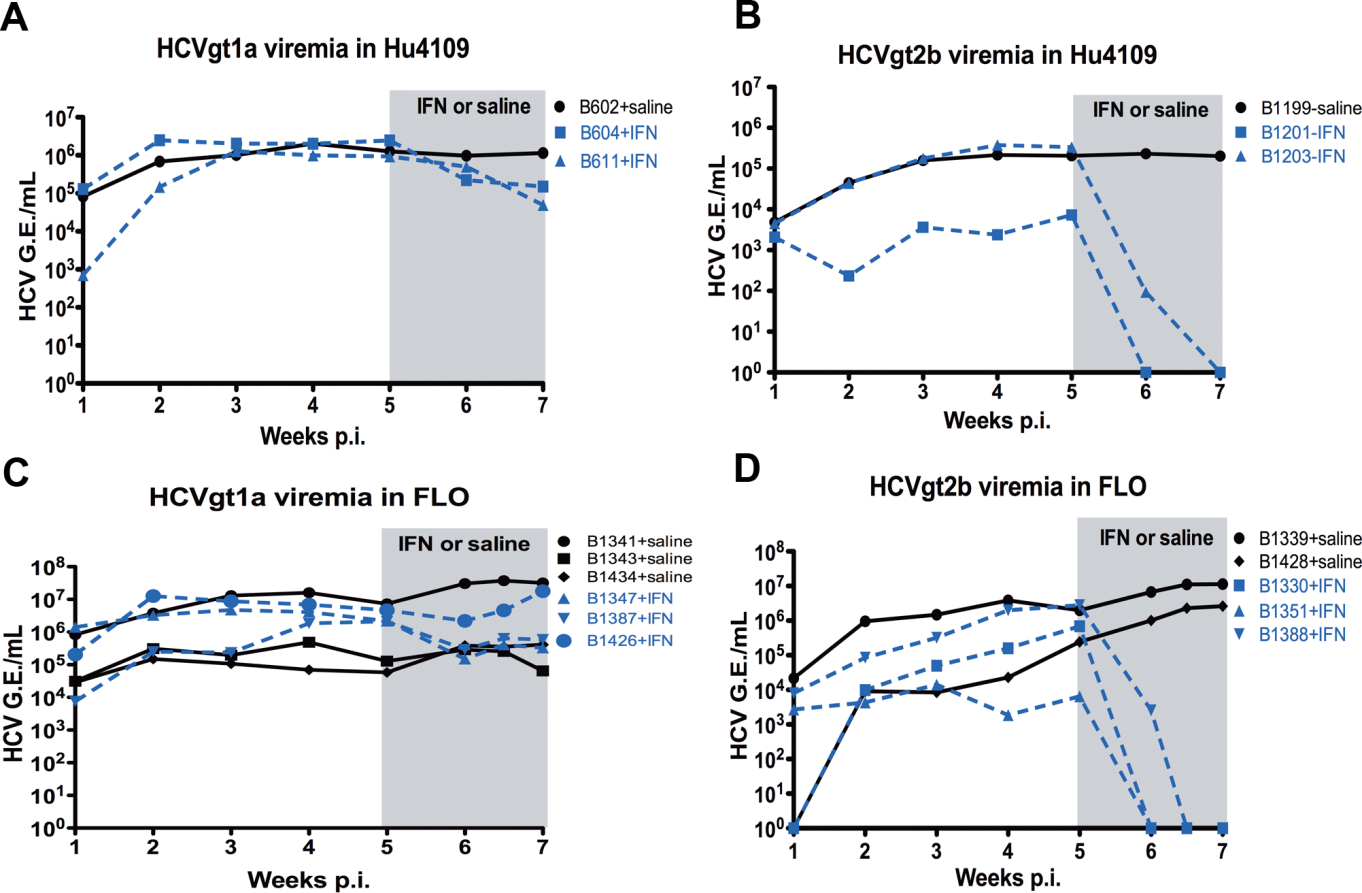
Effect of the poor-responder genotypes of *IL28B/IFNL4* SNPs on HCV responses to IFNα in mice. We infected chimeric mice populated with human hepatocytes from two donors, Hu4109 (**A, B**) and FLO (**C, D**), with HCVgt1a (**A, C**) or HCVgt2b (**B, D**). We administered exogenous human IFNα-2b for 14 days starting at 5 weeks post-infection. We administered saline to control chimeric mice on the same schedule. We collected sera weekly to measure viremia. Results for mice treated with saline are represented by black solid lines and data for mice treated with human IFNα are represented by blue dashed lines. The period of IFNα/saline treatment is shaded in gray.

We performed the experiments described in Fig 1 using chimeric mice transplanted with hepatocytes from donor Hu8063, who had responder alleles at all three SNPs (Table 4). To investigate the impact of host *IL28B*/*IFNL4* polymorphisms on IFNα treatment outcomes in the chimeric mouse model, we produced mice using two other hepatocyte donors, Hu4109 and FLO, with poor responder genotypes at all three SNPs, as listed in Table 4. Chimeric mice produced with hepatocytes from Hu4109 or FLO were infected with the same two HCV strains (Table 1) with different IFN sensitivities, and exogenous human IFNα was administered for 2 weeks. We quantified HCV viremia by RT-qPCR and compared levels before and after human IFNα treatment. If the three SNPs were critical determinants of the outcomes of IFN therapy in these chimeric mice, we hypothesized that the HCV viremia decline would be more rapid in chimeric mice produced with the responder hepatocytes from donor Hu8063 and slower in chimeric mice produced with the poor responder hepatocytes from donors Hu4109 or FLO when these animals were treated with exogenous human IFNα. This hypothesis, however, was not supported by our results, which showed that the IFN-nonresponsive HCVgt1a strain remained nonresponsive to human IFNα treatment in chimeric mice produced with either the *IL28B*/*IFNL4* responder (Hu8063 in Fig 1A) or poor responder genotype hepatocytes (Hu4109 and FLO in Fig 4A and 4C). The IFN-sensitive strain HCVgt2b had similar sensitivities in both sets of chimeric mice produced with the *IL28B*/*IFNL4* poor responder genotype hepatocytes (Fig 4B and 4D). This response was as good as or better than the response of this HCVgt2b strain in chimeric mice produced with hepatocytes of the *IL28B*/*IFNL4* responder genotype as shown in Fig 1B. These results again suggest that viral factors are likely more important determinants of responses to IFN therapy than the host factors, such as *IL28B* and *IFNL4* SNPs in HCV-infected chimeric mice.

**Table 4:** Three lines of chimeric mice populated with three different human hepatocyte donors with distinct *IL28B* and *IFNL4* SNPs.

Hepatocyte donor	*rs12979860*	*rs8099917*	*rs368234815*	Genotype
Hu8063	CC	TT	TT/TT	Responder
Hu4109	TT	GG	ΔG/ΔG	Poor responder
FLO	CT	TG	ΔG/TT	Poor responder

## Discussion

In this study, we evaluated both viral and host factors that have been shown to predict responses of CHC patients to pegIFNα and RBV combination therapy using the SCID/beige-Alb/uPA chimeric mouse model.

Among viral factors, HCV genotype is the strongest predictor of nonresponse to IFN treatments [3,30,31]. In our study, we used two HCV strains belonging to different genotypes, HCVgt1a from a null responder and HCVgt2b from a SVR patient. In infected donor-matched chimeric mice, each HCV strain retained the same IFN sensitivity exhibited in the patient from which it had been isolated. To further demonstrate the critical role of viral factors in determining IFN therapy treatment outcomes, we used additional HCV clinical isolates from patients with variable treatment results to infect chimeric mice and administered human pegIFNα for 4 weeks. We found that although the hepatocyte background in the chimeric mice was different from that in patients, the response of each HCV isolate to IFN therapy in patients was correlated to the response in the HCV-infected chimeric mice treated with pegIFNα. This supports the observation that viral factors are critical determinants of responses to IFN treatment. To further investigate the intrinsic viral factors that determine viral response to IFN treatment, we performed sequencing analysis using the next-generation sequencing technology. In the HCVgt1a virus used in this study, amino acid substitutions, that were reported associated with HCV resistance to IFN therapy in patient studies, were not observed. Despite this, the HCV strain was non-responsive to IFN treatment in the chimeric mice, indicating additional viral intrinsic factor(s) remain responsible for the non-responsiveness.

Data about the molecular mechanism underlying HCV sensitivity to IFNα therapy are limited, but evidence that HCV antagonizes the host IFN response pathway [25,32,33] suggests a hypothesis that the lack of response to IFN therapy by nonresponsive HCV strains may result from their more effective suppression of the host *JAK-STAT* pathway. We tested this hypothesis by measuring ISG transcription levels in HCV-infected human hepatocytes after IFNα treatment. Surprisingly, our results indicate that ISG mRNA levels in chimeric mice infected with the IFN-nonresponsive HCV strain were not lower than those in mice infected with the IFN-sensitive strain, suggesting that both strains have similar effects on the IFN response pathway in the chimeric mouse model. Thus, HCV sensitivity to IFNα therapy is not critically associated with viral interference with host IFN signaling downstream of IFN receptors in this mouse model.

We examined two host factors affecting IFN therapy treatment outcomes: liver ISG gene expression prior to IFN therapy and the hepatocyte donor genotype at the *IL28B/IFNL4* loci.

Evidence from clinical studies shows that upregulation of a subset of ISGs before treatment is strongly associated with nonresponse to exogenous IFN therapy [7,8]. Liver biopsy samples from the patients contain mixtures of several cell types besides hepatocytes. The data thus represent the IFN responses from the mixture of cell types residing within the liver. In the chimeric mouse livers, human hepatocytes are the primary human cell type present. Our results show no significant upregulation of human endogenous type I and III IFNs or ISGs in response to long-term infection by either of the two HCV strains in chimeric mice. This observation suggests that the upregulation of ISGs seen in patient liver biopsy studies may be a result of ISG expression in cell types other than hepatocytes or direct or indirect interactions between hepatocytes and other cell types. The finding that plasmacytoid dendritic cells (pDCs) can sense HCV infected cells and produce robust IFNα provides supporting evidence for the importance of cells other than hepatocytes in this experiment [34,35]. In line with the pDC result, a recent study suggested that Kupffer cells, the residing macrophages in the liver, are a possible source of hepatic IFN in CHC patients and that local IFNβ production by Kuppfer cells may drive ISG set point levels in surrounding hepatocytes [36]. Together, the evidence suggests that induced ISG responses in the livers of CHC nonresponders to IFN therapy result from coordination or cross-talk between hepatocytes and other types of cells, in particularly immune cells, residing in or infiltrating the liver.

On the other hand, our finding that no significant ISG upregulation is induced during long-term HCV infection in chimeric mice was unexpected, as we had previously observed an ISG response in HCV-infected chimeric mice [37]. Perhaps a portion of the chimeric mice used in our previous studies had beige trait leakage, which resulted in increased mouse NK cell function, or the chimeric mice used in the previous study were produced with fresh human hepatocytes isolated from liver tissues taken at the time of donor surgery, which almost certainly would have contained other liver cell types. Both factors could have contributed to the production of an ISG response in our previously reported study [37]. In the current study, the beige trait was confirmed and we used cryopreserved hepatocytes rather than fresh hepatocytes.

Large-scale genome-wide association studies have been used to identify host markers associated with responsiveness to IFN treatment of HCV infection, including two SNPs upstream of the IL28B coding region: *rs12979860* and *rs8099917* [9–11,29]. Recently, a Japanese group found no significant HCV RNA reduction differences in chimeric mice sera between responder and poor-responder IL28B genotypes of host hepatocytes after human IFNα treatment [38]. However, the three HCV inocula the authors used were very similar in terms of their IFN-sensitivities, which may have obscured the effects of the polymorphisms at the IL28B locus. Using a similar chimeric mouse model, another group reported that the responder IL28B genotype was associated with earlier reduction in HCV RNA [39]. In addition, a novel dinucleotide variant controlling *IFNL4*, *rs368234815*, has been shown to be associated with the response outcomes to treatment with pegIFN/RBV [12]. To further clarify the discrepancy in the current literature regarding SNPs *rs12979860* and *rs8099917* as well as to evaluate the potential effect of the new *IFNL4* SNP on HCV response to IFN treatment, we used two HCV isolates with distinct IFN-sensitivities to infect mice produced with hepatocyte donors carrying three different *IL28B/IFNL4* SNPs including both responder and poor-responder IFN response genotypes. We found that the IFN-nonresponsive HCV strain isolated from a null-responder remained insensitive to human IFNα treatment in all three hepatocyte backgrounds, whereas the IFN-sensitive HCV strain was sensitive in all three host backgrounds. These results suggest that the variants of the *IL28B/IFNL4* SNPs in donor hepatocytes had little direct influence on the responses to IFNα treatment under immunosuppressive conditions of the SCID/beige trait in our chimeric mice, which is in agreement with observations by Watanabe *et al*. [38].

There are some limitations to this study. The two major HCV strains used in this study have distinct IFN sensitivities and they belong to genotype 1a and 2b respectively. Currently, HCV genotype is well known as one of the viral factors that influence the patient response to pegIFN/RBV therapy, e.g genotype 2 is more sensitive than genotype 1. However, we believe our finding is important and novel since the response of each HCV strain to IFNα treatment in the mice correlated with their response in the source patient regardless of the viral genotypes and the host background (Table 2). In this study, due to the technical challenges working with the mouse model, three human hepatocyte donors were used to study the impact of three different *IL28B/IFNL4* SNPs on HCV clearance following IFNα treatment, one representing each of the combinations of “responder” alleles, “poor-responder” alleles and the “mixed” alleles. We acknowledge that larger numbers of human hepatocytes from different patients with the various *IL28B/IFNL4* alleles are essential to be able to draw a conclusion about their weight/influence on the differential responses to IFN treatment. However, it is difficult to identify lots of cryopreserved hepatocytes that produce adequate engraftment in the mice. We also note that the next-generation sequencing experiments that were used to study the potential association between HCV sequence variations and viral response to IFN treatment would benefit from a larger sample size.

Despite these weaknesses, by taking advantage of the unique characteristics of the SCID/beige-Alb/uPA chimeric mouse model, we were able to study the contributions of several host factors, including pre-treatment ISG expression level and *IL28B/IFNL4* polymorphisms, and viral factors, including genotype, viral sequence variations and viral blockage of host IFN signaling, separately in the response of HCV infection to human IFNα therapy. We found that intrinsic viral factors were the key determinants of HCV responses to IFN therapy. This finding was not associated with the known amino acid variations located at HCV core and NS5A regions, or with HCV interference with host IFN signaling. Host factors such as polymorphisms at the *IL28B/IFNL4* loci and pre-treatment levels of intrahepatic ISG expression seemed less important in determining the outcomes of IFN therapy in chimeric mice than they were in CHC patients. The more complex interplay between virus and host seen *in vivo* likely reflects contributions from other cell types, notably those of the immune system, which is absent in the chimeric mouse model.

## Supporting Information

S1 Fig Sustained viremia by both HCV strains in mice populated with hepatocytes from a single donor. We infected age-matched mice produced with hepatocytes from a single donor, Hu8063 (IL28 responder genotype as described in Table 4), with two HCV strains: **(A)** HCVgt1a strain in 4 mice, **(B)** HCVgt2b strain in 3 mice. Each sample was quantified in duplicate. G.E stands for Genome Equivalence.
(TIF)

S2 Fig Albumin levels during HCV infection and IFNα treatment in mice populated with Hu8063. Data for chimeric mice treated with saline are represented by black solid lines and results for mice treated with human IFNα are represented by blue dashed lines. The period of IFNα/saline treatment is shaded. Each line represents a single mouse. **(A)** Human albumin levels over the course of infection and human IFNα treatment in chimeric mice infected with HCVgt1a. **(B)** Serum human albumin levels in chimeric mice infected with HCVgt2.
(TIF)

S3 Fig Number of aligned reads along the HCV-1 genome (GI: 22129792) in LOG scale. (A) Aligned read counts of HCVgt1a human plasma (Sample 1 in S2 Table); (B) Aligned read counts of HCVgt1a mouse plasma with saline treatment (Sample 2 in S2 Table); (C) Aligned read counts of HCVgt1a mouse plasma with IFN treatment (Sample 3 in S2 Table).
(TIF)

S4 Fig Number of aligned reads along the HCV-2 genome (GI: 157781212) in LOG scale. (A) Aligned read counts of HCVgt2b human plasma (Sample 4 in S2 Table); (B) Aligned read counts of HCVgt2b mouse plasma with saline treatment (Sample 5 in S2 Table); (C) Aligned read counts of HCVgt2b mouse plasma with IFN treatment (Sample 6 in S2 Table).
(TIF)

S5 Fig Human ISG expression upon IFNα treatment in HCV-infected chimeric mice compared to uninfected controls. All mice were populated with hepatocytes from a single donor, Hu8063. Results are shown as fold-changes relative to the ISG expression in uninfected saline-treated controls. Each column represents a single mouse in the respective treatment group. Increased and decreased expression of specific genes compared to the control group is indicated in red (Fold >1–≥4) and green (Fold <1–≤0.25), respectively; black indicates no change (Fold = 1).
(TIF)

S1 Table List of PCR oligos for real-time PCR assays. (DOCX)

S2 Table Sample list for next-generation deep sequencing analysis. (DOCX)

S3 Table Genome sequence of HCV gt2b strain assembled using SOAPdenovo based on next-generation sequencing results. (DOCX)

S4 Table Summary of amino acid or nucleotide variations in HCVgt1a and derived mouse samples (samples #1–3 in S2 Table). (XLSX)
