# Supplementary figures and images for: Intrinsic Viral Factors Are the Dominant Determinants of the Hepatitis C Virus Response to Interferon Alpha Treatment in Chimeric Mice
Source: PLoS One. 2016 Jan 14;11(1):e0147007. doi: 10.1371/journal.pone.0147007 (PMC4713165; doi:10.1371/journal.pone.0147007)

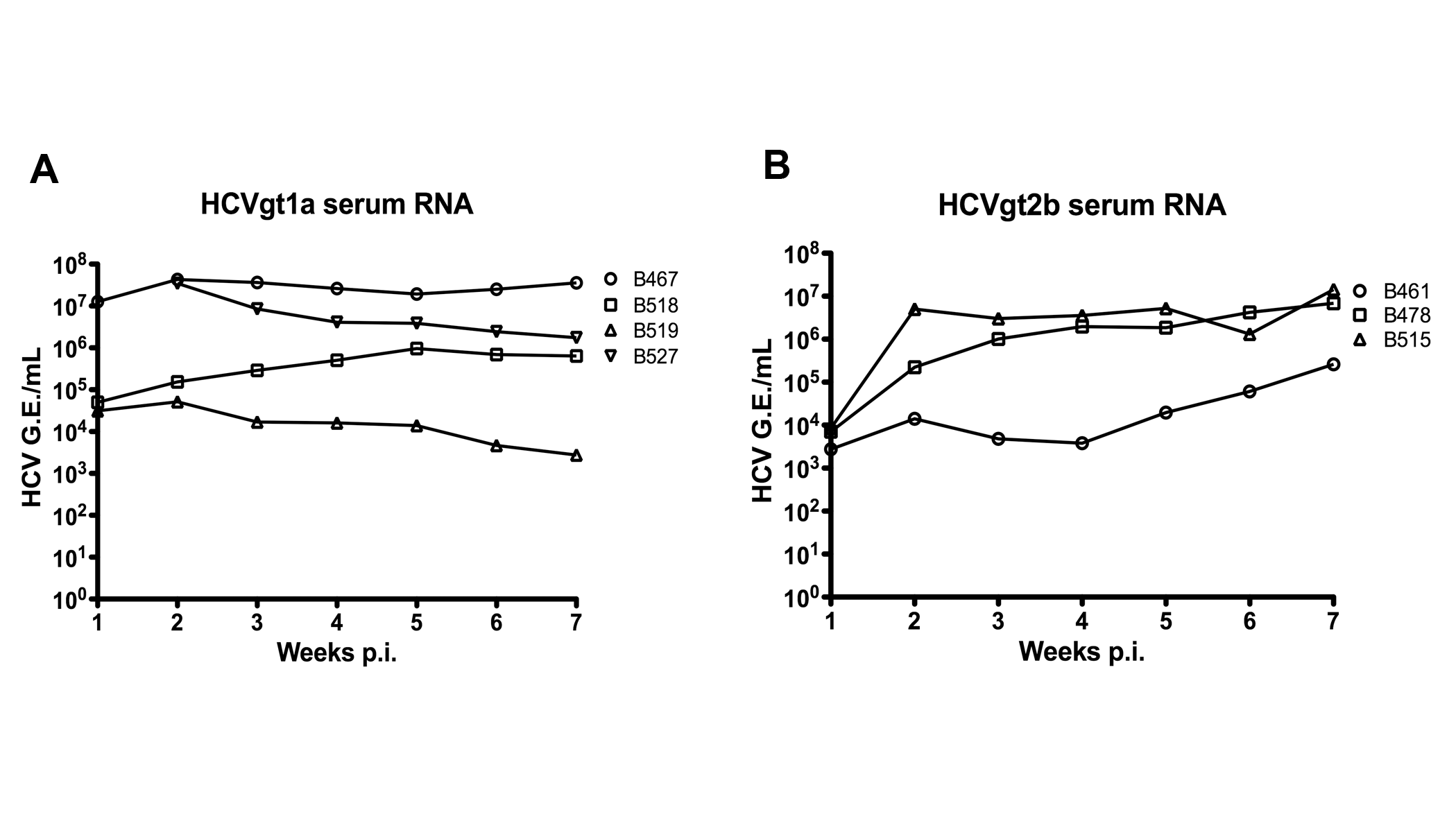

Supplement: S1 Fig — We infected age-matched mice produced with hepatocytes from a single donor, Hu8063 (IL28 responder genotype as described in Table 4), with two HCV strains: (A) HCVgt1a strain in 4 mice, (B) HCVgt2b strain in 3 mice. Each sample was quantified in duplicate. G.E stands for Genome Equivalence. (TIF) [file pone.0147007.s001.tif]

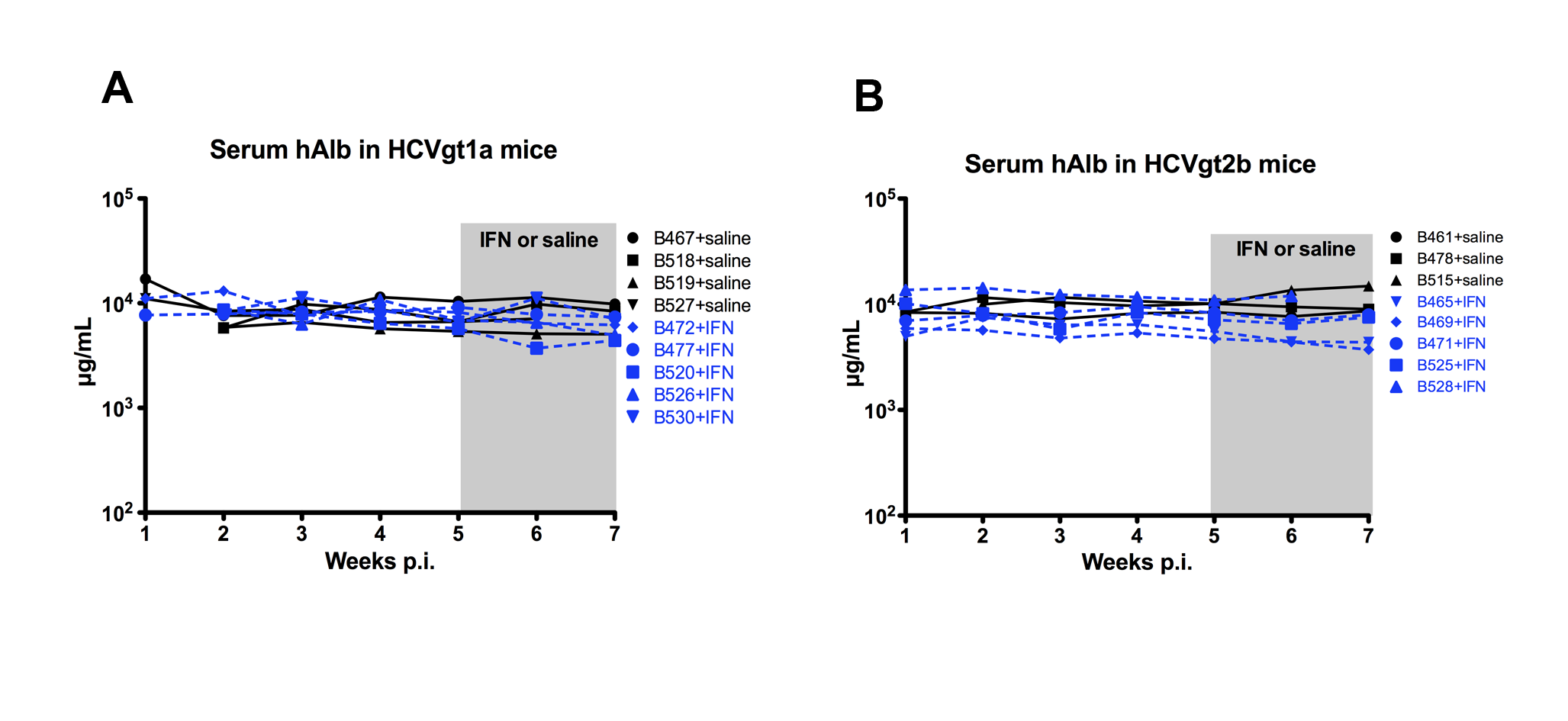

Supplement: S2 Fig — Data for chimeric mice treated with saline are represented by black solid lines and results for mice treated with human IFNα are represented by blue dashed lines. The period of IFNα/saline treatment is shaded. Each line represents a single mouse. (A) Human albumin levels over the course of infection and human IFNα treatment in chimeric mice infected with HCVgt1a. (B) Serum human albumin levels in chimeric mice infected with HCVgt2. (TIF) [file pone.0147007.s002.tif]

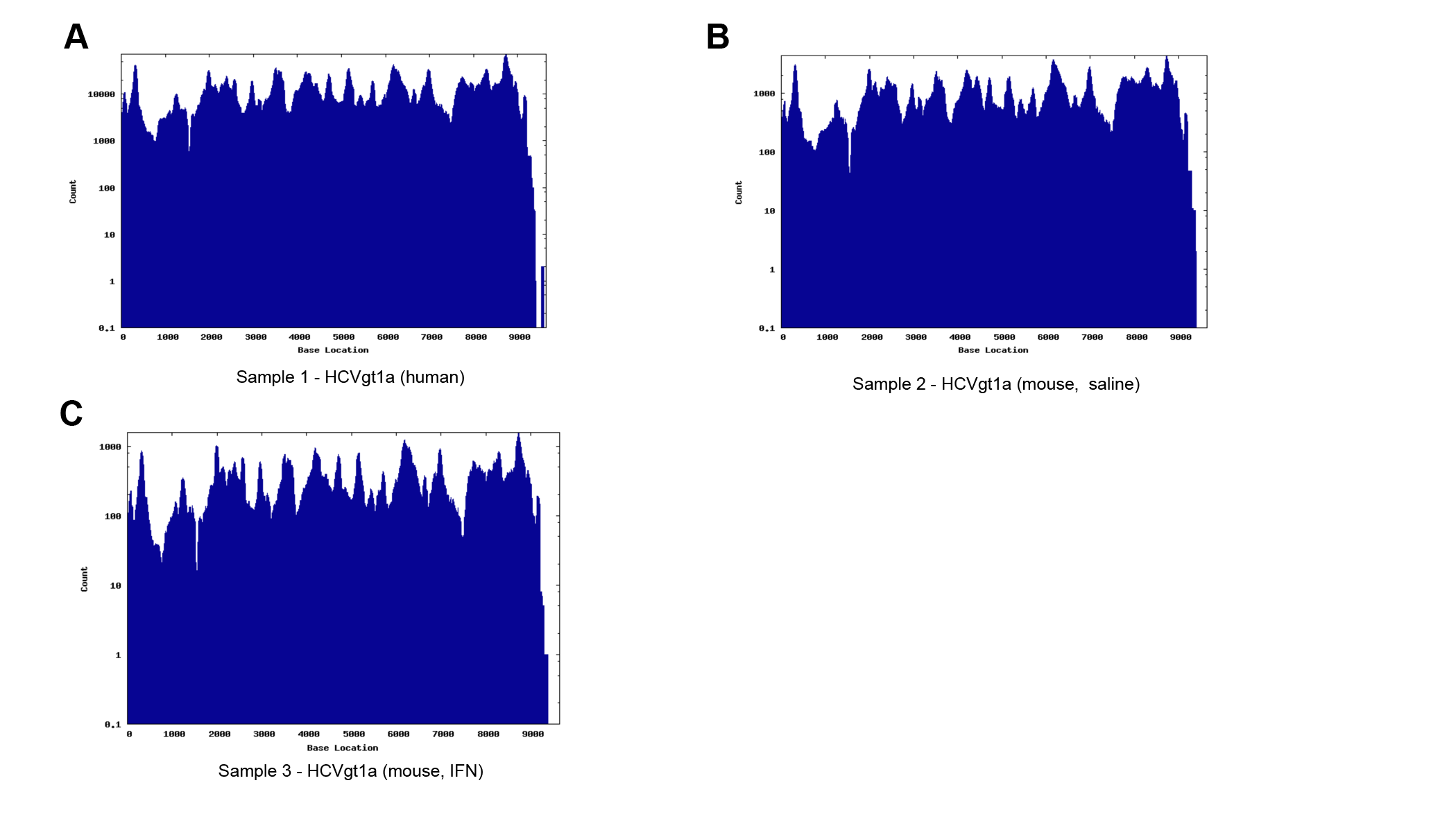

Supplement: S3 Fig — (A) Aligned read counts of HCVgt1a human plasma (Sample 1 in S2 Table); (B) Aligned read counts of HCVgt1a mouse plasma with saline treatment (Sample 2 in S2 Table); (C) Aligned read counts of HCVgt1a mouse plasma with IFN treatment (Sample 3 in S2 Table). (TIF) [file pone.0147007.s003.tif]

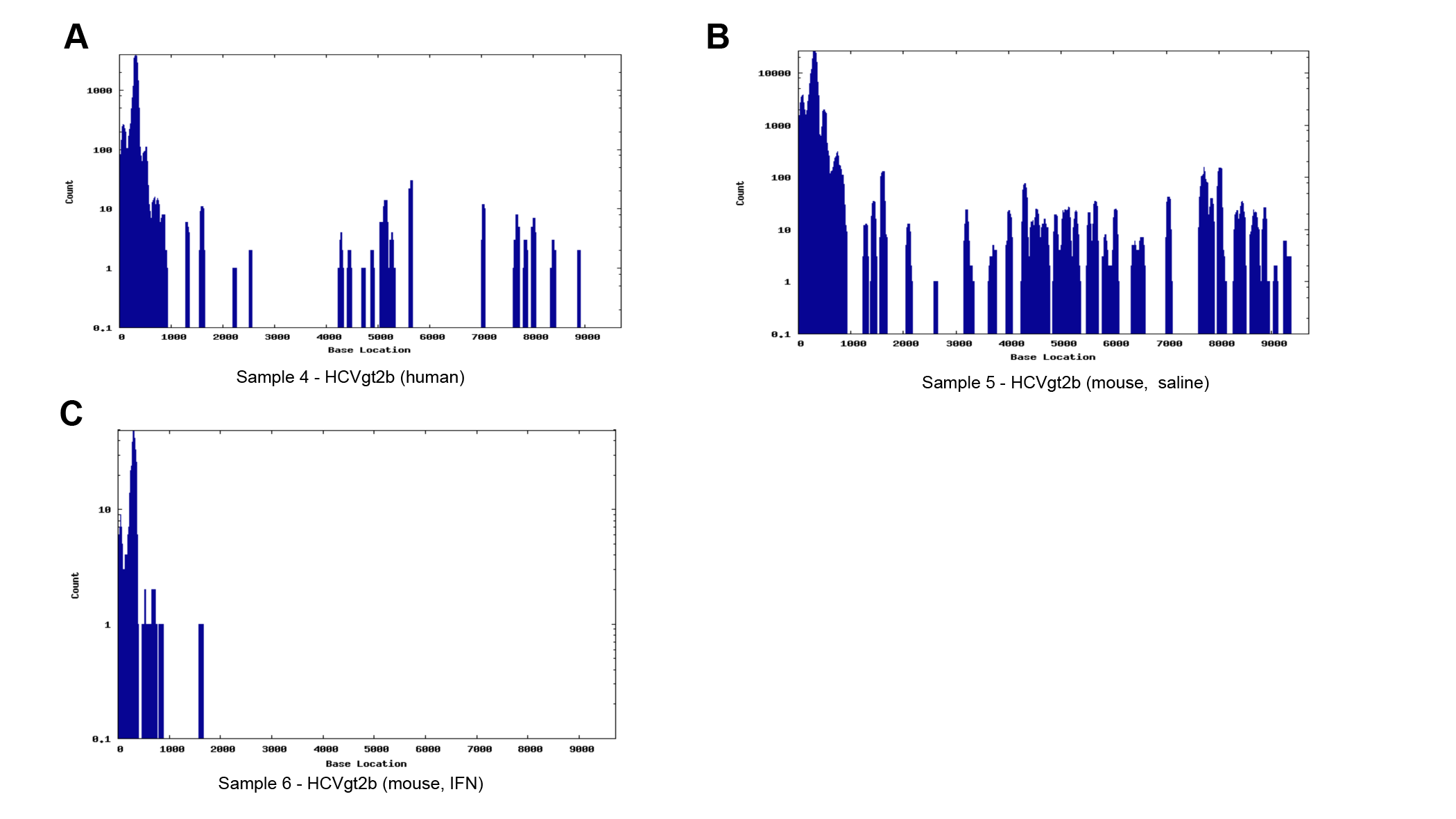

Supplement: S4 Fig — (A) Aligned read counts of HCVgt2b human plasma (Sample 4 in S2 Table); (B) Aligned read counts of HCVgt2b mouse plasma with saline treatment (Sample 5 in S2 Table); (C) Aligned read counts of HCVgt2b mouse plasma with IFN treatment (Sample 6 in S2 Table). (TIF) [file pone.0147007.s004.tif]

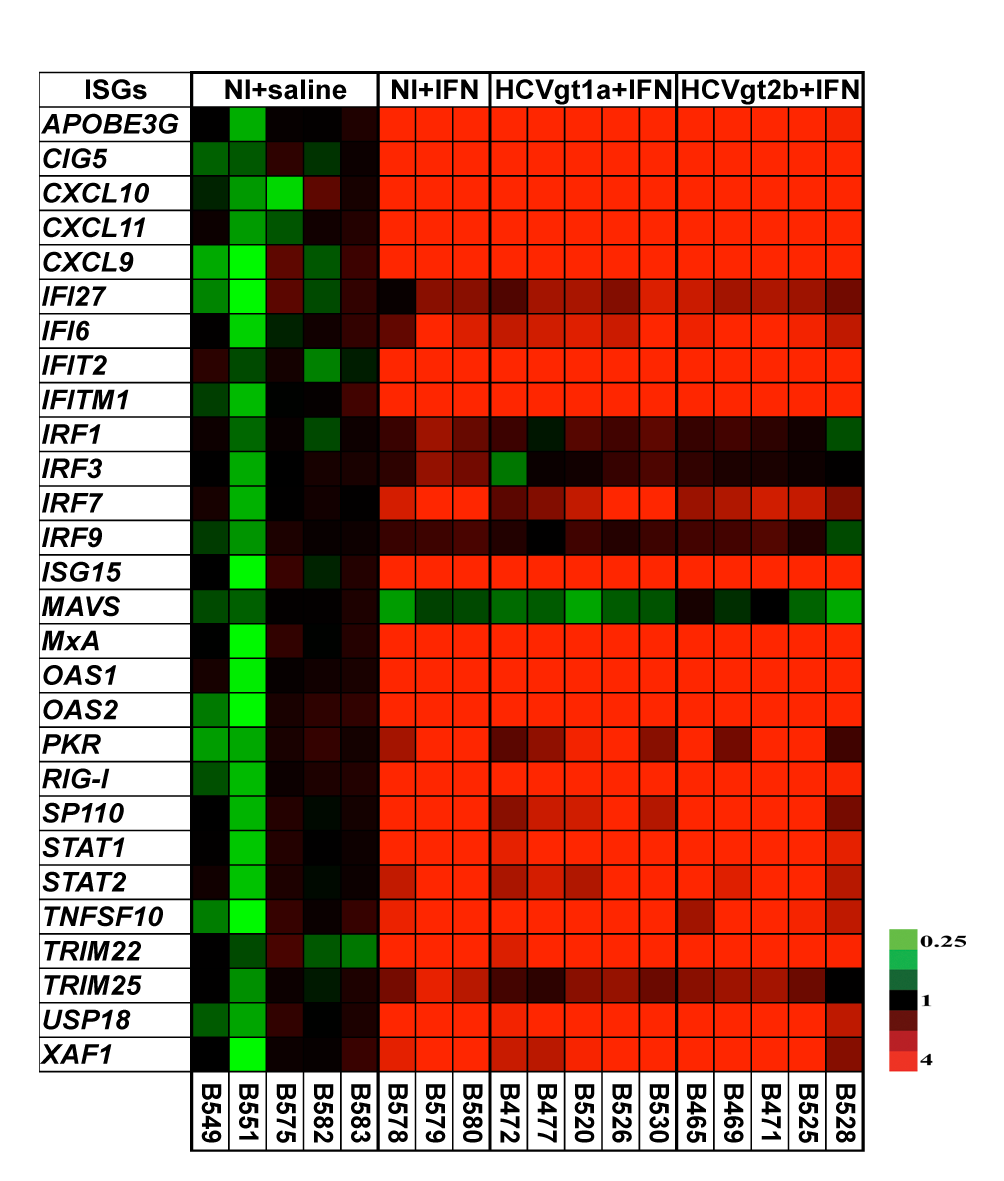

Supplement: S5 Fig — All mice were populated with hepatocytes from a single donor, Hu8063. Results are shown as fold-changes relative to the ISG expression in uninfected saline-treated controls. Each column represents a single mouse in the respective treatment group. Increased and decreased expression of specific genes compared to the control group is indicated in red (Fold >1–≥4) and green (Fold <1–≤0.25), respectively; black indicates no change (Fold = 1). (TIF) [file pone.0147007.s005.tif]
