# Supplementary material for: Intrinsic Viral Factors Are the Dominant Determinants of the Hepatitis C Virus Response to Interferon Alpha Treatment in Chimeric Mice
Source: PLoS One. 2016 Jan 14;11(1):e0147007. doi: 10.1371/journal.pone.0147007 (PMC4713165; doi:10.1371/journal.pone.0147007)
Supplement: S1 Table — (DOCX) [file pone.0147007.s006.docx]

**Suppl. Table 1. List of PCR oligos for real-time PCR assays.**

| **Human Gene** | **Fwd primer (5'->3')** | **Probe (FAM-TAMRA)** | **Rev primer (5'->3')** |
| --- | --- | --- | --- |
| *IFNα1* | AACTCCCCTGATGAATGC | N/A | CTGCTCTGACAACCTCCC |
| *IFNβ* | CAATTTTCAGTGTCAGAAGCTCC | CTGTGGCAATTGAATGGGAGGCTT | AAAGTTCATCCTGTCCTTGAGG |
| *IL28A+B* | GACGCTGAAGGTTCTGGAG | CCACCGCTGACACTGACCCA | ATATGGTGCAGGGTGTGAAG |
| *IL29* | CTGTCACCTTCAACCTCTTCC | CCCATCGGCCACATATTTGAGGTCT | GACGTTCTCAGACACAGGTTC |
| *HPRT 1* | CTTGGTCAGGCAGTATAATCCA | N/A | CAAATCCAACAAAGTCTGGCT |
| *APOBEC3G* | CAACCAGGCTCCACATAAACAC | TTTCCTTGAAGGCCGCCATGCA | GGAATCACGTCCAGGAAGCA |
| *CIG5/Viperin* | AGATGTTTCTGAAGCGAGGA | TGGATTGGTAGAGCGGAAAGTGGA | GCAGACAATGGCAGTTACTC |
| *GIP3/IFI6* | AAGGCCCTGACCTTCAT | AGGAGGACTCGCAGTCGCC | ATTCAGGATCGCAGACCA |
| *HSXIHPAF1* | CTTGAGCACCAGCAGG | TCATAAGGCCAATGAGTGCCAGGA | GCATGTCCAGTTTGCAGA |
| *IFI27* | GTAGTTTTGCCCCTGGC | GACATCATCTTGGCTGCT | TGTGATTGGAGGAGTTGTGGCTGT |
| *IFIT2* | AGGAAGATTTCTGAAGAGTGC | CACTGCAACCATGAGTGAGAACAATAAGAA | GTTCCAGGTGAAATGGCA |
| *IFITM1* | TCCTCATGACCATTGGATTCATC | AGACTGTCACAGAGCCGAATACCA | CCGTTTTTCCTGTATTATCTGTAACATAA |
| *IRF1* | CTGTCGCCATGTGCTGTCA | CAGCACTCTCCCCGACTGGCACA | TGTCCGGCACAACTTCCA |
| *IRF3* | CCCTCACGACCCACATAAAATC | CAGACACCTCTCCGGACACCAATG | CCCAGTAACTCATCCAGAATGTC |
| *IRF7* | GTGAAGCTGGAACCCTGG | AGCGCCAACAGCCTCTATGACG | CCATAAGGAAGCACTCGATGTC |
| *IRF9* | GCCCTACAAGGTGTATCAGTTGCT | CCACCAGGAATCGTCTCTGGCCA | TCGCTTTGATGGTACTTTCTGAGT |
| *ISG15* | TGGTGAGGAATAACAAGGGC | N/A | CAGATTCATGAACACGGTGC |
| *MAVS* | ACCCACAGGGTCAGTTGTATCTACT | TTCTCCTCCTCATCCCCTGGCTTGG | TCACTCTCTGCACCCTGTTTACC |
| *MxA* | ACCTGATGGCCTATCACCAG | N/A | TTCAGGAGCCAGCTGTAGGT |
| *OAS1* | TGTGTGTCCAAGGTGGTAAAGG | CCTCAGGCAAGGGCACCACCCT | CAACCAGGTCAGCGTCAGATC |
| *OAS2* | GGTGAACACCATCTGTGACG | N/A | TGAACCCATCAAGGGACTTC |
| *PKR* | TTAGTGACCAGCACACTCGC | N/A | ATGCCAAACCTCTTGTCCAC |
| *RIG-I* | GGACGTGGCAAAACAAATCAG | ATTGTGATCTCCACTGGCTTTGA | ACACAGGAATGACCCTCCCGGCA |
| *SP110* | CAAAGCGATGAGATCCTGAG | CTTGTCATTGGTCACTGAAGTGCTTCT | CTGAGTCTTCTTCCGCATTC |
| *STAT1* | GTGGAAAGACAGCCCTGCAT | ACTGGACCCCTGTCTTCAAGAC | AACGCACCCTCAGAGGCCGC |
| *STAT2* | ACCAGTTGCTCACTGAGGAGAATATA | GGTAGTAGCACCCAAAAGCTTCA | TGCGCTTCCTCTATCCCCGAATCC |
| *TNFSF10* | TGCGTGCTGATCGTGATCTT | TGCTCCTGCAGTCTCTCTGTGTGGCT | GTACTTGTCCTGCATCTGCTTCA |
| *TRIM22* | GCAGGAGTTTGTGACCAA | CCAAGGGAGCAGTGCAATGGATTT | AGAGGTTCTGTCAGGAGC |
| *TRIM25* | CCGAGGTGGAACTGAACCA | AAGCTGATAAAAGGCATCCACCAGAGCA | TTCAGCTCGTTTTTGAGGTCTATG |
| *USP18* | TGGCCTACTGCCTGCAGAA | TGCAACGTGCCCTTGTTTGTCCA | GGTACAGTTGGGCAGCATCA |
| *rs12979860* | ABI assay ID: C___7820464_10 | | |
| *rs8099917* | ABI assay ID: C___11710096_10 | | |
| *rs368234815* | GCCTGCTGCAGAAGCAGAGAT | Probe AA (VIC): ATCGCAGAAGGCC  Probe ΔC (FAM): ATCGCAGCGGCCC | GCTCCAGCGAGCGGTAGTG |
| *HCV titration* | TCTGCGGAACCGGTGAGTA | CACGGTCTACGAGACCTCCCGGGGCAC | GTGTTTCTTTTGGTTTTTCTTTGAGGTTTAGG |
