# Supplementary material for: Intrinsic Viral Factors Are the Dominant Determinants of the Hepatitis C Virus Response to Interferon Alpha Treatment in Chimeric Mice
Source: PLoS One. 2016 Jan 14;11(1):e0147007. doi: 10.1371/journal.pone.0147007 (PMC4713165; doi:10.1371/journal.pone.0147007)
Supplement: S2 Table — (DOCX) [file pone.0147007.s007.docx]

| **Sample #** | **Sample name** | **Sample input** | **Pools for probe capture** | **HCV seqs**  **(reads per million)** |
| --- | --- | --- | --- | --- |
| 1 | HCVgt1a (human) | 500μL | T1  HCV-1a | 151125 |
| 2 | HCV1a  (mouse, saline) | 600μL |  | 29637 |
| 3 | HCV1a (mouse, IFN) | 750μL |  | 9620 |
| 4 | HCVgt2b (human) | 800μL | T2  HCV-2 | 2870 |
| 5 | HCV2b  (mouse, saline) | 600μL |  | 12354 |
| 6 | HCV2b (mouse, IFN) | 720μL |  | 27 |

**Suppl. Table 2. Sample list for next-generation deep sequencing analysis.**
