# Supplementary material for: Intrinsic Viral Factors Are the Dominant Determinants of the Hepatitis C Virus Response to Interferon Alpha Treatment in Chimeric Mice
Source: PLoS One. 2016 Jan 14;11(1):e0147007. doi: 10.1371/journal.pone.0147007 (PMC4713165; doi:10.1371/journal.pone.0147007)
Supplement: S3 Table — (DOCX) [file pone.0147007.s008.docx]

>HCV-2b-denovo

CGTACAGCCTCCAGGCCCCCCCCTCCCGGGAGAGCCATAGTGGTCTGCGGAACCGGTGAGTACACCGGAATTACCGGAAAGACTGGGTCCTTTCTTGGATAAACCCACTCTATGTCCGGTCATTTGGGCGTGCCCCCGCAAGACTGCTAGCCTAGTAGCGTTGGGTTGCGAACGGCCTTGTGGTACTGCCTGATAGGGTGCTTGCGAGTGCCCCGGGAGGTCTCGTAGACCGTGCATCATGAGCACAAATCCTAAACCTCAAAGAAAAACCAAAAGAAACACAAACCGCCGCCCACAGGACGTCAAGTTCCCGGGTGGCGGTCAGATCGTTGGCGGAGTTTACTTGCTGCCGCGCAGGGGCCCCAGGTTGGGTGTGCGCGCGACGAGGAAGACTTCCGAGCGATCCCAGCCGCGTGGAAGACGCCAGCCCATCCCGAAAGATCGGCGCTCCACCGGCAAGTCCTGGGGAAAGCCAGGATATCCTTGGCCTCTGTACGGAAACGAGGGCTGCGGCTGGGCAGGTTGGCTCTTGTCCCCCCGCGGGTCTCGTCCTACTTGGGGCCCTACTGACCCCCGGCATAGATCACGCAATTTGGGCAAAGTCATCGATACCATTACGTGTGGTTTTGCCGACCTCATGGGGTACATCCCTGTCGTTGGCGCCCCAGTTGGAGGCGTCGCCAGAGCTCTGGCACATGGTGTTAGGGTCCTGGAAGACGGGATAAATTACGCAACAGGGAATTTGCCTGGTTGCTCCTTTTCTATCTTCTTGCTTGCTCTTTTGTCGTGCGTCACAGTGCCAGTGTCTGCAGTGGAAGTCAGGAACATCAGCTCTAGCTACTACGCCACTAATGATTGCTCGAACGACAGCATCACGTGGCAGCTCACTAATGCAGTTCTCCATGTTCCCGGATGCGTCCCATGTGAGAATGATAATGGCACCTTGCGCTGCTGGATACAAGTGACACCTAATGTGGCTGTGAAACATCGTGGCGCACTCACTCATAGCCTGCGAACGCATGTCGACGTGATCGTGGCGGCAGCCACGGTCTGCTCGGCCTTGTACGTGGGAGACGTGTGCGGGGCCGTGATGATTGTATCGCAGGCTTTCATAATATCGCCAGAACGCCACCACTTCACCCAANNNNNNNNNNNNNNNNNNNNNNNNNNNNNNNNNNNNNNNNNNNNNNNNNNNNNNNNNNNNNNNNNNNNNNNNNNNNNNNNNNNNNNNNNNNNNNNNNNNNNNNNNGGTCATATCACCGGCCATCGCATGGCATGGGACATGATGCTAAATTGGTCTCCAACTCTTACCATGATCCTCGCCTACGCTGCTCGCATTCCGGAGCTAGTCCTTGAGATTGTCTTCGGCGGCCACTGGGGTGTGGTGTTTGGCTTGGCCTATTTCTCCATGCAGGGAGCGTGGGCCAAAGTCTTCGCCATCCTCCTCCTTGTCGNNNNNNNNNNNNNNNNNNNNNNNNNNNNNNNNNNNNNNNNNNNNNNNNNNNNNNNNNNNNNNNNNNNNNNNNNNNNNNNNNNNNNNNNNNNNNNNNNNNNNNNNNNNNNNNNNNNNNNNNNNNNNNNNNNNNNNNNAGCAGCGACTCAATTTGATTAACACCAACGGCAGCTGGCATATAAACCGGACCGCCCTCAACTGCAATGACAGCTTGAATACGGGTTTCATCGCTTCCCTGTTTTACACCCACAGCTTCAGCAGCTCTGGCTGCCCCGAGCGCTTGTCTGCCTGCCGCAGGCTGGACGATTTCCGCATCGGGTGGGGAACCTTGGAATACGAGACCAACGTCACCAACGATGACGACATGAGGCCGTACTGCTGGCATTACCCTCCAAAGCCTTGCGGTATCGTTCCGGCTAGGACGGTTTGCGGGCCGGTCTATTGTTTCACTCCCAGTCCTATTGTTGTGGGTACTACTGACAGGCAGGGCGTGCCCACCTATAGATGGGGGGAAAATGAGACCGATGTCTTCATATTAAATAACACAAGACCCCCGCGAGGAGCTTGGTTTGGCTGCACTTGGATGAATGGGACTGGGTTCACTAAGACATGCGGTGCACCACCTTGCCGCATTAGGAGGGATTTCAATAGTACCCTCGATCTGCTGTGCCCTACGGACTGTTTTAGGAAGCACCCAGACGCTACCTATCTTAAGTGCGGAGCAGGGCCTTGGTTGACTCCTAGATGCCTGGTAGATTACCCTTATAGATTGTGGCACTATCCGTGTACTGTGAATTTTACCATCTTCAAGACGCGGATGTTTGTAGGGGGGGTGGAGCATCGGTTCACCGCAGCATGTAACTTCACGCGCGGGGACCCCTGCAGATTGGAAGACAGGGATAGGGGTCAACAGAGTCCGCTACTGCACTCCACCACTGAGTGGGCGGTTCTGCCATGCTCCTTCTCCGACCTACCGGCACTATCTACTGGTCTATTGCACCTCCACCAGAAAATCGTGGACGTACAGTACCTCTANNNNNNNNNNNNAGCTATCACAAGATACCTTGTGAAGTGGGAATGGGTGGTCCTCCTATTCTTGTTGCTGGCAGACGCCAGGATCTGTGCATGCCTTTGGATGCTCATCATACTGGGCCAGGCCTAGGCGGCGCTTGAGAAGCTTATCATCTTGCACTCTGCTAGCGCCGCTAGTGCTAATGGGCCGCTGTGGTTCTTCATCTTTTTTACAGCGGCCTGGTACCTGAAGGGTAGGGTGGTCCCCGCGGCCACATACTCTGTTCTCGGCTTGTGGTCTTTCTTCCTCCTGGTCCTGGCCTTACCACAGCAGGCTTATGCCCTGGACGCCTCTGAGCAAGGGGAACTGGGGCTGGTCCTATTGGCAATCATATCCATCTTTACCCTTACCCCAGCATACAAGACTCTCCTGAGC

CGTTCAGTGTGGTGGCTATCCTATATGCTGGTCTTGGCCGAGGCCCAGATTCAGCAATGGGTTCCCCCCCTGGAGGCCCGGGGGGGGCGTGACGGGATCATCTGGGTGGCTGTCATTCTACACCCACGCCTTGTGTTTGAGATCACGAAATGGTTGTTAGCAGTTCTGGGGCCTGCCCACCTCCTTAGAGCGTCCTTGCTACGGATACCGTACTTTGTGAGGGCCCACGCCCTGCTACGAGTATGCACCATGGTAAGACATCTCGCAGGAGCTAAGTACATCCAGATGCTGCTGATCACCATAGGCAGATTGACCGGCACTTACATCTATGACCACCTCTCCCCTTTATCAACCTGGGCAGCCCAGGGTTTGCGGGACCTGGCAGTCGCCGTGGAGCCTGTGGTGTTTAGCCCAATGGAGAAGAAGGTCTTTGTAGGGGGGGCTGAGACAGTGGCTTGCGGGGACATCCTGCATGGCCTCCCGGTCTCCGCGAGGCTAGGTAGGGAAGTTCTGCTCGGCCCCGCCGATGGCTACACCACGAAGGGGTGGAAGCTCCTAGCCNNNNNTTACTGCTTACACTCAACAGACTCGAGGTCTTCTGGGTGCCATCGTGGTCAGTTTAACGGGCCGCGACAAAAATGAGCAGGCCGGGCATGTCCAGGTTCTGTCCTCTGTTACGCAATCTTTCTTGGGGNNNNNCTATCTCAGGGATCCTCTGGACAGTATATCATGGGGCTGGCAATAAGACCTTGGCCGGCCCTAAAGGGCCAGTCACTCAGATGTACACCAGCGCGGAGGGGGACCTCGTGGGGTGGCCCAGCCCCCCCGGGACTAAGTCCTTGGACCCCTGTACCTGCGGGGCCGTCGACCTCTACCTGGTCACCCGAAACGCTGATGTCATTCCGGTCCGGAGGAAAGGCGACCGGCGGGGTGCACTACTCTCGCCAAGGCCTCTCTCAACCCTCAAAGGGTCATCCGGTGGACCTGTGCTCTGTTCTAGGGGGCACGCCGTGGGCTTGTTCAGAGCGGCCGTGTGCGCCAGGGGTGTGGCCAAGTCTATTGACTTCATCCCTGTTGAATCTCTCGATGTAGCCACGCGGTCGCCCAGCTTTACTGACAACAGCACGCCACCGGCTGTGCCCCAGACATACCAGGTGGGCTATTTGCACGCGCCGACAGGCAGTGGGAAGAGCACCAAGGTCCCTGCCGCGTACGCCAGTCAGGGGTATAAGGTACTTGTACTGAATCCCTCTGTCGCGGCCACACTCGGCTTTGGGGCCTACATGTCCAAAGCCCACGGGATCAACCCCAACATCAGAACTGGAGTACGGACTGTGACCACCGGGGACCCAATCACCTACTCCACTTATGGCAAGTTTCTCGCAGACGGAGGCTGCTCAGCTGGCGCCTATGACGTCATCATATGCGATGAATGCCATGCTGTGGACGCCACCACCATCCTTGGCATTGGAACAGTCCTCGACCAGGCCGAGACCGCAGGTGCCAGGTTAGTGGTCTTGGCCACAGCCACGCCTCCTGGTACAGTNNNNNNNNNNNNNNNNNNNNNNNNNNNNNNNNNNNNNNNNNNNNNNNNNAACAACACCTCATAGTAACATAGAGGAGGTGGCTCTTGGCCACGAAGGCGAGATCCCTTTTTACGGCAAGGCTATCCCCCTAGCTTCCATAAAGGGAGGCAGACACCTGATCTTTTGCCATTCAAAGAAGAAATGCGATGAGCTCGCAGCAGCCCTTCGGAGCATGGGTGTCAATGCCGTTGCTTACTACAGGGGTCTCGACGTCTCCGTTATACCATCTCAGGGGGACGTGGTGGTCGTCGCCACCGATGCCCTAATGACTGGATTCACCGGCGACTTTGACTCTGTCATTGACTGCAACGTTGCAGTCACTCAAATTGTGGATTTTAGCCTAGACCCAACCTTCACCATCACCACCCAAACTGTCCCTCAGGACGCCGTCTCCCGTAGCCAACGTAGAGGGAGAACAGGGAGGGGACGGCTAGGCATCTACAGGTATGTCTCGTCAGGCGAAAGGCCGTCTGGGATGTTCGACAGCGTAGTGCTCTGTGAGTGCTATGATGCCGGGGCGTCCTGGTACGAACTCACGCCTGCTGAGACTACAGTGAGACTCCGGGCTTATTTCAACACGCCCGGCCTGCCCGTCTGCCAAGACCACCTAGAATTCTGGGAGGCGGTGTTTACAGGTCTCACACACATCGATGCCCACTTCCTCTCCCAAACGAAGCAGGGGGGGGACAACTTTGCGTATCTAACGGCCTACCAGGCCACTGTGTGCGCTAGGGCAAAGGCCCCTCCTCCCTCCTGGGACGTGATGTGGAAGTGTCTAACTAGGCTCAAACCTACACTGAATGGCCCTACCCCCCTCCTATACCGCTTGGGTTCCGTGACCAACGAGGTTACCCTGACACACCCCGTGACGAAATATATCGCCACGTGCATGCAAGCTGATCTTGAGATCATGACAAGCACATGGGTTCTGGCAGGGGGGGTGCTGGCCGCCGTGGCAGCTTATTGTCTGGCGACCGGCTGCATCTCCATCATCGGCCGCTTACACCTGAACGATCGGGTAGTTGTGGCCCCTGACAAGGAGATCTTGTACGAGGCCTTTGATGAAATGGAAGAGTGCGCCTCCAAAGCCGCTCTCATCGAGGAAGGGCAGCGGATGGCGGAGATGCTCAAGTCCAAGATACAAGGCCTCTTGCAACAGGCCACAAAACAGGCCCAAGACATACAGCCAGCCATACAGTCATCGTGGCCCAAGCTCGAACAATTTTGGGCCAAGCACATGTGGAACTTCATCAGCGGCATACAGTACCTGGCGGGACTCTCCACTCTACCGGGAAATCCCGCGGTGGCATCAATGATGGCTTTTAGCGCCGCATTGACTAGCCCACTACCCACCAGCACCACCATCCTCTTGAACATTATGGGGGGATGGTTGGCCTCTCAGANNNNNNNNNNNNNNNNNNNNNNNNNNNNNNNNNNNNNNNNNNNNNNNNNNNNNNNNNNNNNNNNNNNNNNNNNNNNNNNNNNNNNNNNNNNNNNNNTTGCCCCCCCCGCCGGAGCCACTGGCTTCGTTGTCAGTGGTCTAGTGGGGGCGGCCGTCGGAAGTATAGGCCTGGGCAAAATACTGGTGGATGTTTTGGCTGGATATGGCGCAGGCATTTCAGGGGCCCTCGTAGCTTTTAAGATCATGAGCGGCGAGAAGCCCACAGTTGAAGATGTTGTAAACCTCCTGCCTGCTATCTTGTCTCCCGGTGCCCTGGTAGTGGGGGTCATCTGCGCAGCAATTTTGCGCCGCCACGTTGGTCAGGGGGAGGGGGCGGTTCAGTGGATGAACAGACTGATCGCCTTCGCCTCCAGGGGAAACCACGTTGCCCCCACTCACTACGTGGCAGAGTCTGATGCCTCTCAGCGCGTAATGCAAGTGCTGAGTTCACTTACAATTACCAGCTTACTCAGGAGACTACATGCCTGGATCACTGAGGATTGCCCAGTCCCGTGCTCGGGGTCTTGGCTCCGGGACATTTGGGATTGGGTCTGTTCCACTCTCACAGACTTCAAGAACTGGCTGTCTTCAAAACTGCTCCCCAAGCTGCCCGGTCTCCCCTTTATCTCTTGCCAAAAGGGGTATAGGGGTGTATGGGCTGGCACGGGAGTCATGACTACTCGGTGTCCGTGCGGGGCAAGCATCTCGGGCCATGTCCGCTTGGGCACCATGAAAATAACAGGCCCGAAGACCTGCTGGAACCTATGGCAGGGGACCTTCCCCATCAATTGTTACACAGAGGGGNNNNNNNNNNNNNNNNNNNNNNNNNNNNNNNNNNNNNNNCCTTGCGTGCCAAAACCCCCTCCTAATTATAAGACCGCAATTTGGAGGGTGGCAGCGTCAGAGTACGTTGAGATCACGCAGCATGGCTCTTTCTCGTACGTAACAGGGTTAACCAATGACAACCTTAAGGTCCCCTGCCAGGTACCGGCCCCAGAATTTTTCTCCTGGGTAGATGGGGTGCAGATACACCGGTTCGCCCCCACTCCGGGTCCTTTCTTTCGGGATGAGGTGACGTTCACCGTAGGTCTCAATTCCTTTGTGGTCGGCTCTCAGCTCCCTTGCGATCCCGAGCCGGACACGGAGGTATTGGCCTCCATGTTGACAGACCCGTCCCACATTACAGCGGAGGCGGCGGCTAGGCGGTTGGCCAGGGGGTCTCCCCCCTCACAGGCCAGCTCTTCAGCGAGCCAGCTCTCCGCCCCGTCCCTGAAGGCCACCTGCACCACCCACAAGACGGCATACGACTGCGACATGGTGGATGCCAACCTCTTCATGGGAGGCGATGTGACCCGGATTGAATCCGACTCTAAGGTGATTGTTCTCGATTCCCTCGATTCCATGACTGAGGTAGAGGACGATCGCGAGCCTTCTATACCATCAGAATACTTGGTCAAGAGGAGAAAGTTTCCACCGGCACTACCTCCCTGGGCCCGTCCAGATTACAACCCTCCCGTACTGGAGACNNNNNNNNNNNNNNNNNNNNNNNNNNNNNNNNNNNNNNNNNNNNNNNNNNNNNNNNNNNNNNNNNNNNNNNNNNNNNNNNNNNNNNNNNNNNNNNNNNNNNNNNNNNNNNNNNNNNNNAGGCTGTGCCCTTCCCCCCACACCTCAAGCGCCAGTGCCTCCACCTCGAAGGCGCCGTGCCAAAGTCCTGACTCAGGACAATGTGGAGGGGGTCCTCAGGGAGATGGCGGACAAAGTGCTCAGCCCTCTCCAAGATTGCAATGACTCCGGTCACTCCACTGGGGCGGACACCGGAGGAGACAGCGCCCAGCAACCCCCCGACGAGACTGCCACTTCGGAGGCGGGATCACTGTCCTCCATGCCTCCCCTTGAGGGAGAGCCGGGAGACCCTGACCTGGAGTTTGAACCGGCTGGATCCACTCCCCCTTCCGAGGGGGAGTGTGAGGTCGTTGATTCAGACTCTAAGTCGTGGTCCACAGTCTCTGATCAGGAGGATTCTGTCATCTGTTGTTCCATGTCATACTCCTGGACGGGGGCCCTCATAACACCATGCGGGCCCGAGGAGGAGAAATTACCAATCAACCCTCTGAGCAATTCGCTCATGCGGTTTCACAACAAGGTGTACTCCACAACCTCGCGGAGTGCCTCTCTGAGGGCNAAGAAGGTGACCTTTGACAGGACACAAGTGCTGGACGCGCACTACGACTCAGTCTTGCAGGACATTAAGCGGGCCGCCTCTAAGGTTAGCGCGAGGCTCCTCTCANTAGAGGAAGCCTGCGCACTGACCCCGCCTCATTCCGCCAAATCACGGTACGGATTCGGGGCAAAAGAGGTGCGCAGCTTATCCAGGAGGGCCATCAACCACATCCGGTCCGTGTGGGAGGACCTCCTGGAAGACCATTGTACCCCAATTGACACAACTATCATGGCCAAAAATGAGGTGTTTTGTATTGATCCCGCTAAAGGTGGGAAAAAGGCAGCCCGCCTCATCGTGTACCCCGACCTCGGGGTCAGGGTGTGCGAAAAAATGGCCCTCTATGACATTGCACAGAAGCTTCCCAAGGCAATAATGGGATCATCCTATGGATTCCAATACTCCCCCGCGGAACGGGTTGATTTTCTCCTCAAGGCTTGGGGAAGTAAGGAGGACCCAATGGGGTTCTCGTATGACACCCGTTGCTTCGACTCAACCGTCACAGAGAGGGACATAAGAACAGAAGAATCCATATATCAGGCTTGTTCCCTGCCCGAGGAGGCCAGAACTGTCATTCATTCGCTCACTGAGAGACTTTACGTAGGAGGGCCCATGATGAACAGCAAGGGGCAATCCTGCGGTTACAGGCGATGCCGCGCGAGCGGCGTCTTCACTACTAGTATGGGAAATACCATGACATGCTACATCAAAGCCCTCGCAGCGTGCAAAGCCGCGGGGATCGTGGACCCCACTATGCTGGTGTGTGGAGACGACCTGGTCGTCATCTCAGAGAGCCAAGGCAACGAGGAGGACGAGCGGAACCTGAGAGCTTTCACGGAAGCTATGACCAGGTATTCAGCCCCTCCCGGCGACCTTCCCAGACCGGAATATGACTTGGAGCTTATAACATCCTGCTCCTCAAACGTATCGGTCTGGGAAGGTCGCCGGGAGNNNNNNNNNNNNNNNNNNNNCACTCCAATTTCCCGAGCTGCTTGGGAAACAGTGAGACACTCCCCTGTCAATTCTTGGCTGGGTAACATCATTCAATACGCCCCTACAATCTGGGTACGG
